# Prospects for HIV control in South Africa: a model-based analysis

**DOI:** 10.3402/gha.v9.30314

**Published:** 2016-06-08

**Authors:** Leigh F. Johnson, Calvin Chiu, Landon Myer, Mary-Ann Davies, Rob E. Dorrington, Linda-Gail Bekker, Andrew Boulle, Gesine Meyer-Rath

**Affiliations:** 1 Centre for Infectious Disease Epidemiology and Research, School of Public Health and Family Medicine, University of Cape Town, Cape Town, South Africa; 2 Health Economics and Epidemiology Research Office, Department of Internal Medicine, School of Clinical Medicine, Faculty of Health Sciences, University of the Witwatersrand, Johannesburg, South Africa; 3 Division of Epidemiology and Biostatistics, School of Public Health and Family Medicine, University of Cape Town, Cape Town, South Africa; 4 Centre for Actuarial Research, University of Cape Town, Cape Town, South Africa; 5 Desmond Tutu HIV Centre, University of Cape Town, Cape Town, South Africa; 6 Center for Global Health and Development, Boston University, Boston, MA, USA

**Keywords:** HIV/AIDS, mathematical model, South Africa

## Abstract

**Background:**

The goal of virtual elimination of horizontal and mother-to-child HIV transmission in South Africa (SA) has been proposed, but there have been few systematic investigations of which interventions are likely to be most critical to reducing HIV incidence.

**Objective:**

This study aims to evaluate SA's potential to achieve virtual elimination targets and to identify which interventions will be most critical to achieving HIV incidence reductions.

**Design:**

A mathematical model was developed to simulate the population-level impact of different HIV interventions in SA. Probability distributions were specified to represent uncertainty around 32 epidemiological parameters that could be influenced by interventions, and correlation coefficients (*r*) were calculated to assess the sensitivity of the adult HIV incidence rates and mother-to-child transmission rates (2015–2035) to each epidemiological parameter.

**Results:**

HIV incidence in SA adults (ages 15–49) is expected to decline from 1.4% in 2011–2012 to 0.29% by 2035 (95% CI: 0.10–0.62%). The parameters most strongly correlated with future adult HIV incidence are the rate of viral suppression after initiating antiretroviral treatment (ART) (*r*=−0.56), the level of condom use in non-marital relationships (*r*=−0.40), the phase-in of intensified risk-reduction counselling for HIV-positive adults (*r*=0.29), the uptake of medical male circumcision (*r*=−0.24) and the phase-in of universal ART eligibility (*r*=0.22). The paediatric HIV parameters most strongly associated with mother-to-child transmission rates are the relative risk of transmission through breastfeeding when the mother is receiving ART (*r*=0.70) and the rate of ART initiation during pregnancy (*r*=−0.16).

**Conclusions:**

The virtual elimination target of a 0.1% incidence rate in adults will be difficult to achieve. Interventions that address the infectiousness of patients after ART initiation will be particularly critical to achieving long-term HIV incidence declines in South Africa.

## Introduction

South Africa has the largest HIV epidemic in the world (1). Despite substantial progress in reducing rates of AIDS mortality (2) and mother-to-child transmission (3), HIV incidence rates remain unacceptably high, with around 1,000 new infections occurring daily (4). There is thus an urgent need to improve upon existing prevention programmes. However, it is not clear which approaches to HIV prevention are likely to have the greatest impact.

**Fig. 1:**
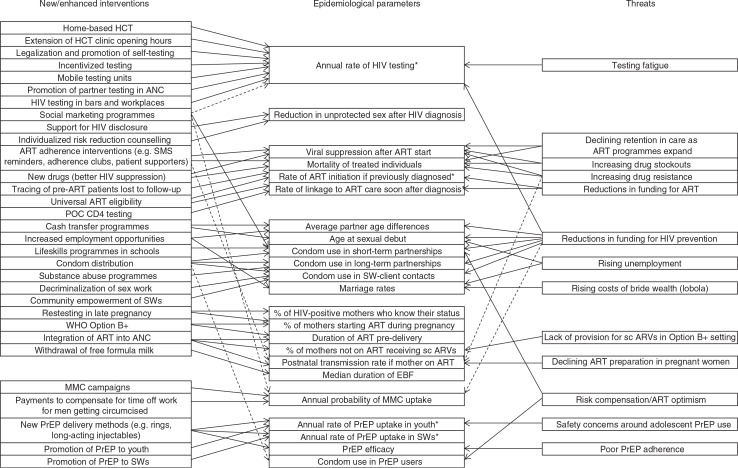
Mapping of effects of interventions and threats on epidemiological parameters in the Thembisa model. Dashed lines represent effects that are less direct or less well-established. *Corresponds to multiple parameters in Table 1. ANC = antenatal care, ART = antiretroviral treatment, EBF = exclusive breastfeeding, EID = early infant diagnosis, HCT = HIV counselling and testing, POC = point of care, PrEP = pre-exposure prophylaxis, sc ARVs = short-course ARVs, SWs = sex workers.

Although many mathematical models have been developed to evaluate the potential impact of individual interventions in South Africa (5–13), to date few have compared the likely impact of a range of intervention combinations (14–17). In most models of combinations of prevention strategies, different interventions are presented as operating independently on different epidemiological parameters, compared to a baseline of ‘no change’. However, the reality is often more complex, with multiple interventions affecting the same epidemiological parameter, and various factors threatening to reverse previous gains (Fig. 1). There may be diminishing marginal returns when multiple interventions affect the same parameter, and the implicit assumption that parameters will not deteriorate from their baseline levels can be overly optimistic (18). Rather than focus on specific intervention programmes, it may be useful to consider which of the more proximal epidemiological parameters/indicators are most important in driving HIV incidence. The primary objective of this study is therefore to evaluate which epidemiological parameters are likely to be most critical to the future reduction of HIV incidence in South Africa and, based on this, to identify priorities for HIV prevention strategies.

A secondary objective is to evaluate South Africa's potential to achieve the ‘virtual elimination’ targets that have been suggested for both horizontal and mother-to-child transmission, as well as the 90-90-90 targets proposed by UNAIDS (19). As proposed by Granich et al. (20), virtual elimination of HIV transmission in adults is defined as an HIV incidence rate in 15–49-year-olds of less than 0.1% per annum. In the context of mother-to-child transmission, virtual elimination is defined as a transmission risk of less than 5% for perinatal and postnatal transmission combined (21). The UNAIDS target is to achieve by 2020 90% of the HIV-positive population diagnosed, 90% of HIV-diagnosed individuals on antiretroviral treatment (ART), and 90% of ART patients virally suppressed (19). An evaluation of South Africa's potential for virtual elimination is needed because although there has been much rhetoric around this goal, most models of HIV in South Africa suggest that it is unlikely that ART alone would achieve this (22). It remains unclear whether multiple interventions in combination could make virtual elimination a plausible scenario.

## Methods

*Thembisa* (meaning ‘give hope’ in Xhosa and Zulu) is a mathematical model that was developed as a combined demographic and epidemiological simulation tool for South Africa. A full description of the model is provided in the Supplementary file. In brief, the model is deterministic and compartmental, dividing the population into a large number of cohorts and simulating the change in cohort sizes over time, starting in 1985. Cohorts are defined in terms of demographic characteristics (age and sex) and behavioural characteristics (marital status, sexual experience, and propensity for commercial sex and concurrent partnerships). In addition, cohorts are defined in terms of level of exposure to HIV prevention programmes, with individuals classified according to their HIV testing history (23), current receipt of pre-exposure prophylaxis (PrEP)/microbicides, and (in the case of men) circumcision status. HIV-positive individuals are further classified according to their level of engagement in HIV care (undiagnosed, diagnosed but untreated, and treated) and CD4 cell count. Three relationship types are modelled: short-term, long-term (marital), and sex worker–client relationships. HIV transmission probabilities per sex act are assumed to depend on the relationship type, the HIV stage and sex of the HIV-positive partner, and rates of condom usage, the latter being assumed to depend on age, relationship type, calendar year, and level of engagement in HIV care (if the individual is HIV positive). HIV transmission probabilities from treated individuals depend on assumed rates of viral suppression, viral load distributions, and the effect of viral load on HIV infectivity (24).

The model considers two forms of mother-to-child transmission: perinatal (at/before birth) and postnatal (through breastfeeding). In both cases, the transmission probability is assumed to depend on the mother's stage of HIV disease and the type of antiretroviral prophylaxis received by the mother and infant. The monthly probability of postnatal transmission is also assumed to depend on the type of breastfeeding.

In adults, ART can be initiated either soon after HIV diagnosis (if the individual is ART-eligible) or at a later time. The model allows for changes in ART eligibility criteria over time and assumes that the probability of ART initiation in recently tested adults depends on the context in which testing takes place (highest in antenatal settings and symptomatic patients), whereas the rate of ART initiation in previously diagnosed adults depends on their CD4 cell count.

The model incorporates historic data on numbers of HIV tests performed, numbers of individuals starting ART, numbers of medical male circumcision (MMC) operations, and rates of uptake of prevention of mother-to-child transmission (PMTCT) services. The model has been calibrated to HIV prevalence data from national household surveys and antenatal clinic surveys, as well as sex worker prevalence studies, using a Bayesian model fitting procedure (further detail is provided in the Supplementary file). For the purpose of this analysis, the parameters considered in the Bayesian analysis have been fixed at their posterior means, so that the focus is limited to future sources of epidemiological uncertainty.

Based on literature reviews and expert consultations, epidemiological parameters likely to be affected by future interventions and threats were identified (Fig. 1). For each epidemiological parameter, probability distributions were specified to represent the range of uncertainty around the parameter value, given the range of intervention options and their likely characteristics, and given possible threats to programme success (Table 1). A justification for the chosen range of uncertainty is provided for each parameter in the Supplementary file. Beta distributions are used to represent the uncertainty regarding proportions and other parameters that are bounded between 0 and 1, while gamma distributions are used to represent uncertainty regarding rates and other parameters that are strictly positive but not subject to any upper bound. Weibull distributions are used to represent uncertainty regarding the likely time to the introduction of new interventions, in years from mid-2015 (in all cases a median of 10 years and a shape parameter of 0.55 is used, which means that there is a 30% chance that the intervention will be introduced within 3 years and a 50% chance that the intervention will be introduced by mid-2025). For most of the parameters for which a baseline period is specified in Table 1, the parameter value sampled relates to the period 5 years after the baseline year, with the parameter values in the intervening 4 years being linearly interpolated between the baseline value and the ultimate value to represent a gradual phasing in of the interventions or environmental changes over the 5-year period.

**Table 1:** Parameters included in uncertainty analysis

Parameter		Baseline value^a^	Baseline period^a^	Prior distribution	Prior mean, standard deviation	95% CI
1.	HIV counselling and testing					
	1.1 Annual rate of first-time HIV testing in non-pregnant HIV-negative women at age 25	0.30	2011/12	Gamma (17.36, 69.4)	0.25, 0.06	0.15–0.38
	1.2 Ratio of male HCT uptake to female HCT uptake at age 25 (HIV negative, non-pregnant)	0.68	2011/12	Gamma (94.4, 138.8)	0.68, 0.07	0.55–0.82
	1.3 Time to the introduction of home-based HCT (in years after 2014/15)	–	–	Weibull (0.195, 0.55)	33.1, 65.1	0–21+^b^
	1.4 Annual rate of HIV testing through home-based HCT	–	–	Gamma (5.44, 15.56)	0.35, 0.15	0.12–0.70
	1.5 Time to the introduction of intensified counselling for positives (in years after 2014/15)	–	–	Weibull (0.195, 0.55)	33.1, 65.1	0–21+^b^
	1.6 Coverage of intensified risk reduction counselling (of diagnosed HIV positives)	–	–	Uniform (0, 1)	0.50, 0.29	0.03–0.98
	1.7 Reduction in unprotected sex following intensified counselling in HIV-positive adults	–	–	Beta (2.083, 2.083)	0.50, 0.22	0.10–0.90
2.	Adult ART					
	2.1 Percentage of patients starting ART with CD4 <200 who are virally suppressed (VL <400 copies/ml)	0.77	2012/13	Beta (10.5, 3.136)	0.77, 0.11	0.52–0.94
	2.2 Time to the introduction of universal ART eligibility (in years after 2014/15)	–	–	Weibull (0.195, 0.55)	33.1, 65.1	0–21+^b^
	2.3 Time to the introduction of POC CD4 testing (in years after 2014/15)	–	–	Weibull (0.195, 0.55)	33.1, 65.1	0–21+^b^
	2.4 Fraction starting ART immediately after diagnosis if POC CD4/universal ART available	0.40	2012/13	Beta (13.8, 9.2)	0.60, 0.10	0.40–0.79
	2.5 Mean time to women starting ART if ART is not started at time of diagnosis (in months)	20	2013/14	Gamma (5.06, 0.281)	18.0, 8.00	5.9–36.7
3.	Behaviour change					
	3.1 Average partner age difference in non-marital relationships	3.00	2011/12	Gamma (9, 3)	3.00, 1.00	1.37–5.25
	3.2 Ratio of marriage rates in 2016/17 to those in 2011/12	1.00	2011/12	Gamma (25, 25)	1.00, 0.20	0.65–1.43
	3.3 Ratio of sexual debut rates in 2016/17 to those in 2011/12	1.00	2011/12	Gamma (25, 25)	1.00, 0.20	0.65–1.43
	3.4 Odds of condom use in marital relationships (relative to 1998)	1.78	2011/12	Gamma (14.97, 8.41)	1.78, 0.46	1.00–2.79
	3.5 Odds of condom use in non-marital relationships (relative to 1998)	3.14	2011/12	Gamma (4.89, 1.56)	3.14, 1.42	1.00–6.47
	3.6 Odds of condom use in SW–client relationships (relative to 1998)	3.80	2011/12	Gamma (3.84, 1.01)	3.80, 1.94	1.00–8.44
4.	Prevention of mother-to-child transmission					
	4.1 Rate of retesting in late pregnancy	0.45	2011/12	Beta (9.02, 3.01)	0.75, 0.12	0.48–0.94
	4.2 Fraction of newly diagnosed pregnant women who start ART prior to delivery	0.75	2011/12	Beta (49.73, 5.525)	0.90, 0.04	0.81–0.96
	4.3 Proportionate increase in mean duration of ART prior to delivery (relative to pre-2010)	0.50	2011/12	Gamma (25, 35.71)	0.70, 0.14	0.45–1.00
	4.4 Relative rate of short-course antiretroviral uptake if long-term ART not started prior to delivery	1.00	2012/13	Uniform (0, 1)	0.50, 0.29	0.03–0.98
	4.5 Relative infectivity of HIV-positive women on long-term ART (breastfeeding)	0.20	2011/12	Beta (3.00, 12.00)	0.20, 0.10	0.05–0.43
	4.6 Median duration of EBF in months, if EBF is initiated	2.00	2010/11	Gamma (16, 4)	4.00, 1.00	2.29–6.00
	4.7 Fraction of mothers discontinuing EBF who stop breastfeeding completely	0.30	2010/11	Beta (5.16, 29.25)	0.15, 0.06	0.05–0.28
5.	Medical male circumcision					
	5.1 Annual probability of MMC uptake in men in non-marital relationships	0.15	2013/14	Beta (1.880, 4.387)	0.30, 0.17	0.04–0.68
6.	Pre-exposure prophylaxis					
	6.1 Effectiveness of PrEP	–	–	Beta (1.27, 1.9)	0.40, 0.24	0.03–0.88
	6.2 Proportionate reduction in condom usage in PrEP users	–	–	Beta (0.8, 7.2)	0.10, 0.10	0.00–0.37
	6.3 Time to the introduction of PrEP for sex workers (in years after 2014/15)	–	–	Weibull (0.195, 0.55)	33.1, 65.1	0–21+^b^
	6.4 Time to the introduction of PrEP for youth aged 15–24 (in years after 2014/15)	–	–	Weibull (0.195, 0.55)	33.1, 65.1	0–21+^b^
	6.5 Annual rate at which sex workers adopt PrEP if it is available	–	–	Gamma (2.25, 7.50)	0.30, 0.20	0.04–0.80
	6.6 Annual rate at which sexually active youth adopt PrEP if it is available	–	–	Gamma (2.25, 7.50)	0.30, 0.20	0.04–0.80

a No baseline value or baseline period is specified for parameters that relate to interventions not yet introduced in South Africa.

b A delay of more than 20 years means that the intervention is not introduced over the period considered in this analysis; hence the upper limit of the distribution is irrelevant. ART=antiretroviral treatment, EBF=exclusive breastfeeding, HCT=HIV counselling and testing, MMC=medical male circumcision, PrEP=pre-exposure prophylaxis, POC=point of care, SW=sex worker, VL=viral load.

Latin hypercube sampling was used to sample 1,000 sets of parameters from these distributions (25). For each of the 1,000 parameter sets, the model was run to the year 2035, and average HIV incidence rates over the 2015–2035 period were calculated, for the 15–49 age group and for age- and sex-stratified sub-groups. Average mother-to-child transmission rates over the 2015–2035 period were also calculated, including all perinatal and postnatal transmission in the numerator and including all mothers who are HIV positive at delivery or seroconvert during breastfeeding in the denominator. Correlation coefficients were calculated to determine the strength of the associations between the parameters and the outcomes of interest. A multivariate linear regression model was also fitted to predict the change in the log-transformed incidence rate per unit change in each epidemiological parameter, using a forward stepwise selection procedure. In addition to this multivariate analysis, one-way sensitivity analyses were performed to assess the effect of changing each parameter from its median value to the upper and lower limits of the corresponding 95% confidence interval (shown in the final column of Table 1).

The Thembisa model used in this analysis was programmed in C++. Further information on the model is available from www.thembisa.org (see version 2.4), and the code used in the current paper is available from the authors on request.

## Results

**Fig. 2:**
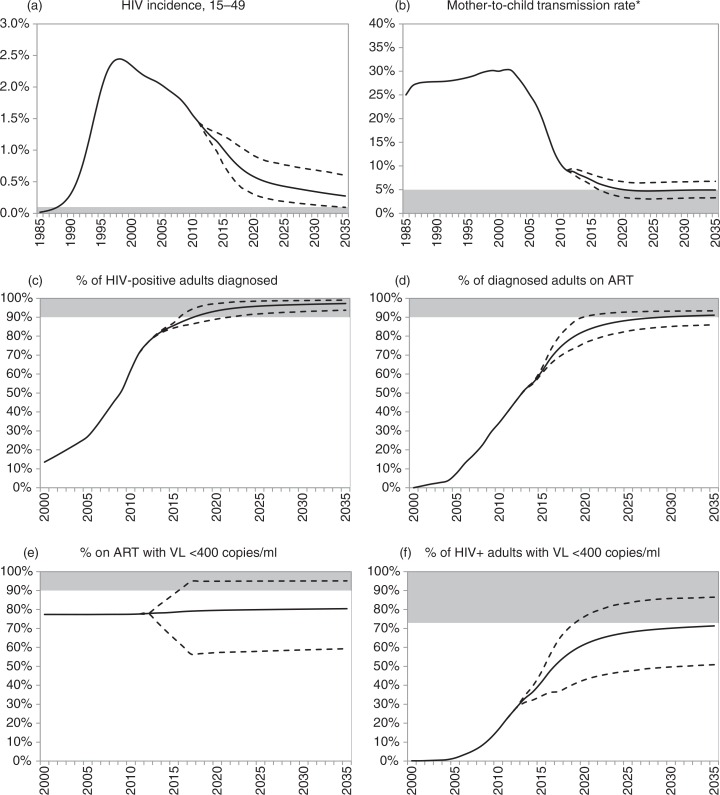
South African HIV incidence trends and progress towards the 90-90-90 targets. Solid lines represent averages from 1,000 simulations; dashed lines represent 95% confidence intervals (2.5 and 97.5 percentiles of distribution of model outputs). Shaded areas represent virtual elimination targets (panels a, b) and 90-90-90 targets (panels c through f). *Denominator is the number of births to HIV-positive mothers plus the number of mothers who seroconvert while breastfeeding, and numerator includes all cases of perinatal and postnatal transmission. ART = antiretroviral treatment. VL=viral load.

The model estimates that in the 2011–2012 period the incidence rate in 15–49-year-olds was 1.4% (Fig. 2a). This number is projected to decline to 0.29% by 2035 (95% CI: 0.10–0.62%), and the virtual elimination threshold of 0.1% is reached before 2035 in only 2% of simulations. Combined perinatal and PMTCT rates in South Africa are estimated at 9.1% in the 2011–2012 period (Fig. 2b). As a result of reductions in adult HIV incidence and further improvements in the PMTCT programme, the transmission rate is projected to decline to an average of 5.2% in 2019–2020 (95% CI: 3.7–6.9%), and to remain relatively stable after 2020; the virtual elimination threshold of 5% is reached by 2035 in 55% of scenarios.

It is highly likely that the first 90% target will be met, with the expected fraction of HIV-positive adults diagnosed increasing to 93% (95% CI: 89–97%) by 2020 (Fig. 2c). However, meeting the second and third 90% targets will be more challenging: the fraction of HIV-diagnosed adults on ART is expected to increase to 83% (95% CI: 77–90%) by 2020 (Fig. 2d), and the assumed uncertainty regarding future viral suppression rates implies that 80% (95% CI: 57–95%) of ART patients are likely to be virally suppressed in 2020 (Fig. 2e). In only 0.4% of simulations are all three 90% targets met, and the expected fraction of HIV-positive adults who are on ART and virally suppressed in 2020 (63%, 95% CI: 44–79%) is also expected to fall short of the 73% level that would be achieved if all three 90% targets were met (Fig. 2f). Details of the epidemiological parameters in the scenarios in which the virtual elimination and 90-90-90 targets are met are presented in the Supplementary file (Supplementary Table 3.3).

**Fig. 3:**
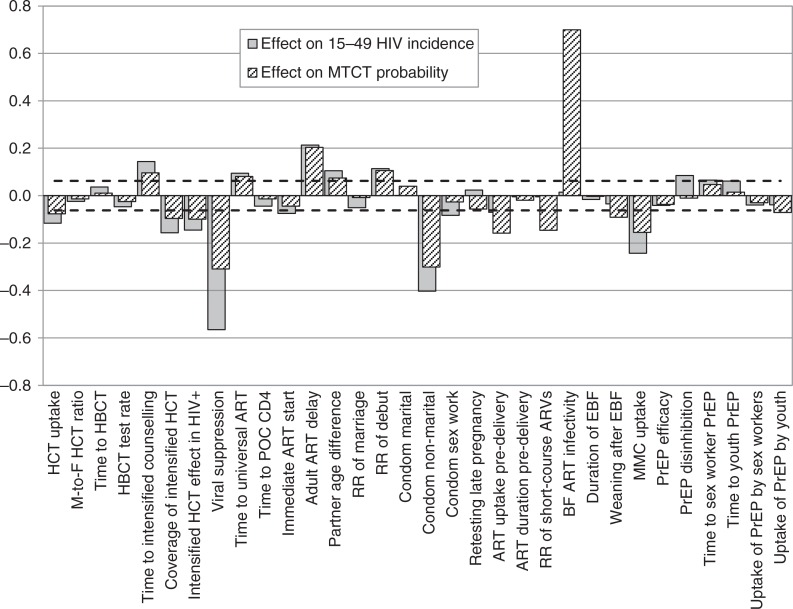
Correlation coefficients between epidemiological parameters and HIV incidence measures over the 2015–2035 period. Zero represents no association, and the interval between the dashed lines represents correlation coefficients that are not significantly different from zero. A positive value represents a positive association between the parameter of interest and future HIV incidence. ART = antiretroviral treatment, BF = breastfeeding, EBF = exclusive breastfeeding, HBCT = home-based counselling and testing, HCT = HIV counselling and testing, MMC = medical male circumcision, POC = point of care, PrEP = pre-exposure prophylaxis, RR = relative rate.

Figure 3 shows the correlation coefficients for the associations between each of the epidemiological parameters listed in Table 1 and the average HIV incidence rate measured over the 2015–2035 period. In adults aged 15–49, HIV incidence is most strongly correlated with the rate of virological suppression after initiating ART (*r*=−0.56), the level of condom use in non-marital relationships (*r*=−0.40), the year of introducing intensified risk-reduction counselling for HIV-positive adults (*r*=0.29), the uptake of MMC (*r*=−0.24), the year of introducing universal ART eligibility (*r*=0.22), and the average delay to ART initiation in previously diagnosed, ART-eligible adults (*r*=0.21). Other significant parameters include the year of introducing PrEP for youth (*r*=0.19), the coverage and effectiveness of intensified risk reduction counselling in HIV-diagnosed adults (*r*=−0.16 and *r*=−0.15 respectively), the year of introducing home-based HIV counselling and testing (HCT) (*r*=0.14), the annual rate of HCT uptake (*r*=−0.12), the rate of sexual debut (*r*=0.11), partner age differences (*r*=0.10), the level of risk compensation if PrEP were introduced (*r*=0.08), the level of condom use in sex worker–client relationships (*r*=−0.08) and the rate of ART initiation during pregnancy (*r*=−0.07). All other epidemiological parameters were not significantly associated with adult HIV incidence in the univariate analysis.

Results were broadly consistent in the multivariate analysis, though a number of additional parameters became significant when other sources of variation were controlled for (Table 2). For each 10% increase (absolute) in the proportion virally suppressed, in adults starting ART with CD4 <200 cells/µl, the future adult HIV incidence is expected to reduce by a factor of 0.857 (95% CI: 0.851–0.862). Equivalently, a 3.3% increase in this rate of viral suppression would achieve a 5% relative reduction in adult HIV incidence rates.

**Table 2:** Multivariate analysis of epidemiological parameters affecting HIV incidence in 15–49-year-olds (2015–2035)

Unit change	RR (95% CI) of HIV incidence per unit change	*p*	Change required to achieve a 5% incidence reduction
10% increase in viral suppression (absolute)	0.857 (0.851–0.862)	<0.001	3.31%
10% increase in odds of condom use (ST partners), relative to 2011/12	0.972 (0.970–0.973)	<0.001	17.8%
10-year reduction in time to intensified counselling	0.916 (0.908–0.924)	<0.001	−5.82
10-year reduction in time to universal ART eligibility	0.924 (0.916–0.931)	<0.001	−6.45
10% increase in annual MMC uptake (absolute)	0.965 (0.961–0.968)	<0.001	14.2%
1-year reduction in ART delay following diagnosis	0.909 (0.899–0.918)	<0.001	−0.53
10-year reduction in time to youth PrEP	0.932 (0.925–0.940)	<0.001	−7.32
10% increase in coverage of intensified risk reduction counselling for HIV-diagnosed	0.983 (0.981–0.986)	<0.001	0.300
10-year reduction in time to HBCT	0.954 (0.947–0.962)	<0.001	−11.0
10% increase in effectiveness of intensified risk reduction counselling for HIV-diagnosed (absolute)	0.981 (0.978–0.984)	<0.001	7.0%
1-year reduction in partner age difference (ST partners)	0.968 (0.962–0.975)	<0.001	−1.59
10% increase in odds of condom use (SW–client relationships), relative to 2011/12	0.994 (0.993–0.995)	<0.001	87.4%
10% increase in efficacy of PrEP (absolute)	0.990 (0.987–0.993)	<0.001	50.5%
10% increase in annual HCT uptake in youth (absolute)	0.961 (0.950–0.972)	<0.001	12.8%
10% increase in rate of marriage, relative to 2011/12	0.989 (0.986–0.992)	<0.001	46.1%
10% increase in annual PrEP uptake by youth (absolute)	0.989 (0.986–0.993)	<0.001	47.3%
10% decrease in rate of sexual debut, relative to 2011/12	0.992 (0.988–0.995)	<0.001	−62.7%
10% increase in fraction starting ART immediately after diagnosis (absolute)	0.987 (0.981–0.994)	<0.001	40.5%
10% increase in annual HBCT uptake (absolute)	0.995 (0.990–1.000)	0.030	–a
10% increase in male-to-female HBCT uptake ratio (absolute)	0.990 (0.980–1.000)	0.040	50.1%

Parameters are ordered according to statistical significance. Only parameters with *p* values <0.05 were included in the final model.

a It is not possible to achieve a 5% incidence reduction based only on changing this parameter.

ART=antiretroviral treatment, HBCT=home-based counselling and testing, HCT=HIV counselling and testing, MMC=medical male circumcision, PrEP=pre-exposure prophylaxis, ST=short-term, SW=sex worker.

Many of the parameters that significantly affect adult HIV incidence are also significantly associated with average mother-to-child transmission probabilities (Fig. 3), most notably the rate of virological suppression after initiating ART (*r*=−0.31) and the level of condom use in non-marital relationships (*r*=−0.30). In addition, the average mother-to-child transmission probability is significantly associated with the relative risk of transmission through breastfeeding when the mother is receiving ART (*r*=0.70), the rate of ART initiation during pregnancy (*r*=−0.16), the relative rate of short-course antiretroviral prophylaxis in mothers who do not receive ART (*r*=−0.15) and the rate of weaning after exclusive breastfeeding (*r*=−0.09).

The results of the one-way sensitivity analyses were consistent with the results of the multivariate sensitivity analysis (Supplementary file). Correlation coefficients were also mostly similar across age and sex categories (Supplementary Table 3.4). MMC uptake is significantly associated with reduced HIV incidence in men and women of all ages, although the expected reductions are greatest in young men.

## Discussion

This analysis suggests that for the purpose of reducing future heterosexual and mother-to-child transmission of HIV in South Africa, the most important epidemiological parameter to target will be the infectiousness of patients receiving ART. This will mean promoting adherence interventions such as adherence clubs, patient supporters, and SMS contact (26–29) and possibly newer drugs that more effectively suppress HIV (30) and have fewer side effects (Fig. 1). It may also involve ‘return to care’ programmes, such as those piloted in Malawi (31), and community-supported models of care to improve retention (32). This may be particularly important for women during the postnatal period, when loss to follow-up rates are particularly high (33, 34) and there is a high risk of vertical transmission through breastfeeding. The level of unprotected sex after HIV diagnosis is also a highly influential parameter, which suggests that more should be done to support the intensified risk reduction counselling programmes that have been piloted in South Africa (35, 36).

Although many previous modelling studies have illustrated the potential importance of ‘test and treat’ approaches (20, 22), our simulations suggest that the timing of universal ART eligibility does not rank as highly as a number of other epidemiological parameters. This is because relatively high rates of HIV diagnosis and ART uptake have already been achieved in South Africa, and further increases can be expected in the near future even in the absence of further changes to existing screening and treatment strategies (23). Our results suggest that the focus of current ART policy debates needs to broaden, with less emphasis on ART eligibility criteria and substantially more emphasis on programmes to maximise viral suppression. The model baseline assumptions for the 2011–2012 period suggest a 77% rate of virological suppression (<400 copies/ml), which indicates that there is still substantial scope for improvement. Increasing this rate to 90% would achieve a roughly 18% reduction in HIV incidence rates (Table 2). Levels of ART coverage in other southern African countries are similar to those in South Africa (1), but levels of virological suppression are mostly unknown, as most other countries in the region have not monitored viral load historically. As lack of viral load monitoring is probably associated with poorer virological suppression (37), these model results strongly support the introduction of viral load monitoring in those countries that have not yet introduced this, in line with WHO guidelines (38).

The model predicts a 55% chance that South Africa will meet the 5% target for mother-to-child transmission by 2035, given current interventions. Previous modelling work has shown that an increasingly high proportion of mother-to-child transmission in South Africa is from women who have recently seroconverted, especially during the breastfeeding period (39). This explains the strong associations that we observed between future mother-to-child transmission rates and the parameters that have traditionally been considered important only as drivers of adult HIV incidence. It is therefore important that PMTCT programmes consider not only the risks to the infants of HIV-positive mothers, but also the potential HIV risks to HIV-negative mothers, who have a high chance of transmitting HIV to their infants if they acquire HIV during late pregnancy or while breastfeeding (40). Interventions to increase ART initiation during pregnancy (e.g. through integration of ART services into antenatal clinics) will also be important (41). However, consistent with data from Botswana (42), our model suggests that the introduction of WHO Options B and B+ could potentially be increasing the proportion of mothers who receive no antiretrovirals antenatally, and mother-to-child transmission rates are sensitive to the assumed change in access to short-course antiretroviral prophylaxis.

This analysis suggests that MMC is likely to be another important HIV prevention strategy for South Africa. As in other southern African countries, the prevalence of male circumcision has historically been low (5) and MMC uptake has been modest (43). Consistent with other mathematical modelling studies, our model shows that women would benefit indirectly from reduced rates of HIV incidence in their male partners (44). Although there is concern that circumcised men may engage in more unprotected sex if they regard themselves as protected against HIV, none of the studies that have followed circumcised males after the completion of the MMC trials have found evidence of risk compensation (45–47).

This analysis suggests that future HIV incidence rates in South Africa may not be strongly influenced by the introduction of PrEP in sex workers but are likely to be influenced by the introduction of PrEP among youth in the general population. This inconsistency might be explained by differences in rates of condom usage in different risk groups and the potential for risk compensation. Other modelling studies have shown that when condom usage is already very high (as is the case in sex worker–client relationships in most of sub-Saharan Africa) (1), the benefit of introducing PrEP is likely to be small, and the benefit may well be offset by reductions in condom use (48–50). In contrast, when condom usage is relatively modest (as in girls with limited ability to insist on condom use), the likely gains from PrEP are more substantial. If PrEP were made available to particular high risk groups, it would be important to monitor trends in condom usage in those risk groups to ensure that PrEP does not have a negative net effect on HIV transmission rates.

Our results also point to the need for renewed emphasis on condom usage, particularly in non-cohabiting relationships. In other African countries, where marriage tends to be more common (51), more emphasis on condom use in marital relationships may be appropriate. It is less clear if other forms of behaviour change are likely to have a material impact. These results suggest that reductions in partner age differences and rates of sexual debut would have modest effects on overall HIV incidence rates. Cash transfer interventions, which significantly reduce age-disparate relationships (52, 53) and rates of sexual debut (52, 54) could be important in achieving HIV incidence reductions among youth.

This analysis has a number of limitations. Although the probability distributions chosen to represent the ranges of uncertainty around key parameters were based on published evidence, the choice was to some extent subjective, which affects the strength of the correlation in the sensitivity analysis. Certain key populations (men who have sex with men, people who inject drugs, and serodiscordant couples) were not included in the model, either because they are not believed to contribute substantially to overall HIV transmission in South Africa (55–57) or because of limitations of the model structure. Several sources of uncertainty were not considered when projecting the model to 2035: uncertainty regarding the baseline conditions, uncertainty regarding potential new technologies (e.g. HIV vaccines, gene therapy, and functional cures), and uncertainty regarding potential viral evolution (58, 59). Forecasts up to 2035 are thus more uncertain than the 95% confidence intervals suggest and need to be treated with some degree of caution.

Although it is likely that there will be substantial reductions in HIV incidence over the next two decades, this should not be taken as an excuse for complacency. Without substantial investments in programme improvements it is unlikely that virtual elimination of HIV transmission in adults will be achieved by 2035 or that the 90-90-90 targets will be reached by 2020. Many of the model parameters that we have highlighted as being important (such as viral suppression on ART and levels of condom use) are parameters that should *already* have been the focus of prevention and treatment programmes over the last decade, and it is disappointing that there has been little evidence of progress in these areas (60). There needs to be renewed emphasis on ‘getting the basics right’, and the allure of new prevention technologies should not detract from the need to improve existing programmes.

## Supplementary Material

Prospects for HIV control in South Africa: a model-based analysis
