# Supplementary material for: Prospects for HIV control in South Africa: a model-based analysis
Source: Glob Health Action. 2016 Jun 8;9:10.3402/gha.v9.30314. doi: 10.3402/gha.v9.30314 (PMC4901512; doi:10.3402/gha.v9.30314)
Supplement: Prospects for HIV control in South Africa: a model-based analysis [file GHA-9-30314-s001.pdf]

Abridged supplementary material

Table of Contents

1. Justification for prior distributions....4

1.1 HIV counselling and testing (HCT)....4

1.1.1 Annual rate of first-time HIV testing in non-pregnant HIV-negative women aged 25....4

1.1.2 Ratio of male HCT uptake to female HCT uptake at age 25....5

1.1.3 Time to the introduction of home-based HCT....5

1.1.4 Annual rate of HIV testing through home-based HCT....5

1.1.5 Time to the introduction of intensified counselling for positives....6

1.1.6 Coverage of intensified risk reduction counselling (of diagnosed HIV-positives)...7

1.1.7 Reduction in unprotected sex following intensified counselling in HIV-positive adults....7

1.2 Antiretroviral treatment (ART)....7

1.2.1 Fraction of patients starting ART with CD4 <200 who are virally suppressed....7

1.2.2 Time to the introduction of universal ART eligibility....10

1.2.3 Time to the introduction of point-of-care (POC) CD4 testing....10

1.2.4 Fraction starting ART immediately after diagnosis if POC CD4/universal ART available....11

1.2.5 Mean time to women starting ART if ART is not started at time of diagnosis....11

1.3 Behaviour change parameters....13

1.3.1 Average partner age difference in non-marital relationships....13

1.3.2 Ratio of marriage rates in 2016/17 to those in 2011/12....14

1.3.3 Ratio of sexual debut rates in 2016/17 to those in 2011/12....14

1.3.4 Odds of condom use in marital relationships (relative to 1998)....15

1.3.5 Odds of condom use in non-marital relationships (relative to 1998)....17

1.3.6 Odds of condom use in SW-client relationships (relative to 1998) ... 17

1.4 Prevention of mother-to-child transmission (PMTCT) ... 18

1.4.1 Rate of retesting in late pregnancy ... 18

1.4.2 Fraction of newly-diagnosed pregnant women who start ART prior to delivery ... 18

1.4.3 Proportionate increase in mean duration of ART prior to delivery ... 19

1.4.4 Relative rate of short-course ARV uptake if long-term ART not started prior to delivery ... 20

1.4.5 Relative infectivity of HIV-positive women on long-term ART (breastfeeding) ... 21

1.4.6 Median duration of exclusive breastfeeding ... 21

1.4.7 Fraction of mothers discontinuing EBF who stop breastfeeding completely ... 22

1.5 Medical male circumcision ... 22

1.6 Pre-exposure prophylaxis (PrEP) ... 23

1.6.1 Effectiveness of PrEP ... 24

1.6.2 Proportionate reduction in condom usage in PrEP users ... 24

1.6.3 Time to the introduction of PrEP for sex workers ... 25

1.6.4 Time to the introduction of PrEP for youth aged 15-24 ... 25

1.6.5 Annual rate at which sex workers adopt PrEP if it is available ... 25

1.6.6 Annual rate at which sexually active youth adopt PrEP if it is available ... 26

2. Statistical analysis ... 26

2.1 Calibration step 1: Model fitting to adult HIV prevalence data ... 26

2.2 Calibration step 2: Model fitting to paediatric HIV prevalence data ... 27

2.3 Uncertainty analysis: determinants of future HIV incidence trends ... 28

3. Additional results ... 29

3.1 One-way sensitivity analyses ... 29

3.2 Inclusion of uncertainty in baseline conditions ... 33

3.3 Inclusion of correlation between epidemiological parameters ... 34

3.4 Characteristics of scenarios in which targets are met ... 36

3.5 Differences by age and sex..39

References...41

1. Justification for prior distributions

The sections that follow provide the justification for each of the prior distributions referred to in the main text. The prior distributions are presented in the same order as presented in Table 1 of the main text.

1.1 HIV counselling and testing (HCT)

The model of HCT is described in detail elsewhere [1]. Briefly, the population aged 10 and older is divided into three HIV testing history groups (never tested, previously tested negative and previously tested positive). Three types of HIV testing are modelled: testing in antenatal clinics, testing of HIV patients with opportunistic infections (OIs), and testing for other reasons. A base rate of testing is specified in each year, which corresponds to women aged 25 who are non-pregnant and asymptomatic, and the effects of sex and age are incorporated as multiplicative adjustments, while the effects of pregnancy and HIV status are incorporated as additive adjustments.

1.1.1 Annual rate of first-time HIV testing in non-pregnant HIV-negative women aged 25

In calibrating the model to historic data, the average estimate for the rate of first-time testing, in non-pregnant HIV-negative women aged 25, is 0.3 in 2011/12 [1]. South Africa aims to maintain roughly stable annual numbers of HIV tests in the public health sector after 2013, with the target set at 10 million HIV tests per annum, similar to the number of 9.9 million tests performed in 2011/12 [2]. However, there may have been some reduction in numbers of HIV tests in recent years; numbers of HIV tests performed among 15-49 year olds in public health facilities have been reported at 9.0 million in 2012/13 [2] and at 6.7 million in 2013/14 [3]. This could be a reflection of testing fatigue, or reductions in resources to support HCT campaigns. On the other hand, there are a number of ways in which HCT uptake could be increased in future. Do *et al* [4], for example, show that four social marketing programmes in South Africa have had a significant impact on HCT uptake, and estimate that for each unit increase in exposure to these programmes, the odds of HIV testing in 2011/12 was increased by 3% (or 6.7% if accounting for indirect effects). Taking into account that the average exposure score over 2009-2012 was 4.16 and the theoretical maximum score was 9, this suggests a maximum increase of 37% ($1.067^{(9 - 4.16)} - 1$) in the odds of HIV testing if social marketing of HCT were fully scaled up. The introduction of self-testing kits could also lead to increases in the uptake of HIV testing [5]. To represent the uncertainty regarding future trends in the base rate, we assign a beta distribution with a mean of 0.25 and a standard deviation of 0.06 to represent the rate that applies from 2016 onwards. The 2.5 and 97.5 percentiles of this distribution are 0.15 and 0.38 respectively; the former represents a worst case scenario in which the reductions in HCT uptake over the 2011-2014 period continue over the next two years, at the average annual rate of 13% observed; the latter represents an

optimistic scenario in which the odds of HIV testing in 2011/12 is increased by 37% (which is equivalent to a 30% increase in the rate of testing).

1.1.2 Ratio of male HCT uptake to female HCT uptake at age 25

Although the base rate of HIV testing in women is estimated to have increased in the decade up to 2010/11, the relative rate of male HIV testing at age 25 is estimated to have declined from 0.83 in 2002/03 to 0.68 in 2010/11 [1]. This may be because the HCT campaigns of recent years have been conducted mainly through public health facilities, which are attended mostly by women. Recent national surveys suggest that the male-to-female testing ratio may have continued to decline in the post-2010 period [6]. On the other hand, it is possible that new HCT interventions might lead to increases in HIV testing in men specifically. For example, incentivized HCT programmes through mobile HCT clinics may be important in reaching unemployed men who often do not attend fixed health facilities [7]. Mobile HCT is associated with relatively high rates of male participation in urban South Africa, with proportions of male clients ranging from 48-61% (median 52%), in comparison to proportions of 40-50% (median 43%) in fixed health facilities [8-10]. A recent systematic review identified a number of other potential strategies for increasing male uptake of HCT, including promotion of testing of partners of women attending antenatal clinics, offering HIV testing in bars, and promotion of testing of partners of newly-diagnosed individuals [11]. Changing clinic opening hours may also make HCT more accessible to employed men [12]. To represent the uncertainty around the future values of the $B_0(t)$ parameter, we assign a gamma prior to the parameter value in 2017/18. This distribution has a mean of 0.68 (assuming on average no change from the baseline) and a standard deviation of 0.07. The 2.5 and 97.5 percentiles of this distribution are 0.55 and 0.82 respectively. The lower bound corresponds to a worst case scenario, in which the male-to-female testing ratio continues to decline at the same rate that it has over the 2002-2010 period. The upper bound corresponds to an optimistic scenario in which the ratio returns to the levels estimated in 2002.

1.1.3 Time to the introduction of home-based HCT

In the period after 2015, the model also allows for potential additional increases in HIV testing due to home-based counselling and testing (HBCT). It is unclear whether HBCT is likely to be introduced in South Africa in future, and we have therefore modelled the introduction of HBCT in the same way as other new interventions, assigning a Weibull distribution with a median of 10 years and a shape parameter of 0.55 to represent the uncertainty around the time to the introduction of HBCT (in years after 2015).

1.1.4 Annual rate of HIV testing through home-based HCT

Although a number of African studies have shown that HBCT is highly acceptable [13], few studies have reported on the fraction of the total population that is reached and tested through HBCT campaigns. In a randomized trial conducted in a South African community [14], the fraction of the population who reported having ever tested for HIV increased from 31% to 69% over a 14-month period, suggesting an annual testing rate of 0.69 (-ln((1-0.69)/(1-

0.31))×12/14), if it is optimistically assumed that all testing that occurred during the 14-month period was due to the home-based testing programme. Lower rates of testing might be expected in field settings, where fewer resources are likely to be committed to the intervention than in a randomized trial. We assign a gamma distribution to represent the uncertainty around the π parameter. This distribution has a mean of 0.35 and a standard deviation of 0.15, with 2.5 and 97.5 percentiles at 0.12 and 0.70 respectively. The mean and standard deviation have thus been chosen so that the upper bound corresponds to what we might optimistically expect if conditions similar to those in the randomized trial prevailed, while the lower bound reflects minimal uptake of HBCT.

1.1.5 Time to the introduction of intensified counselling for positives

The model assumes that in the period up to 2015, people who have been diagnosed HIV-positive reduce their frequency of unprotected sex by 46% [15-18]. In the period after 2015, it is possible that certain interventions could lead to greater reductions in unprotected sex following diagnosis. For example, Cornman *et al* [19] found that in a South African healthcare setting, HIV-positive patients who were randomized to receive special risk reduction counselling reported roughly 90% less unprotected sex with partners of negative or unknown HIV status compared to HIV-positive patients who did not receive the counselling. The same authors found that when repeating the intervention in a larger trial, the reduction in unprotected sex with partners of negative/unknown status was slightly more modest, 59% after 18 months [20]. However, in another South African trial involving a similar intervention, there was no reduction in unprotected sex in women who received special counselling over a 3-month period [21]. In other African settings, facilitated disclosure of HIV status to sexual partners has been shown to significantly increase levels of disclosure [22], which is likely to also be important in reducing levels of unprotected sex in HIV-diagnosed individuals. Given the intensive nature of these counselling interventions, it is unlikely that they would reach all HIV-diagnosed individuals.

To model the effect of HIV diagnosis on sexual risk behaviour, we define $\delta(t)$ to be the reduction in unprotected sex in HIV-diagnosed individuals, relative to undiagnosed individuals, in year t. The symbol δ_0 represents the reduction associated with current South African programmes and policies (0.46), while δ_1 represents the additional reduction that might be expected in future if new counselling and facilitated disclosure interventions were introduced. In mathematical terms,

$$\delta(t) = \begin{cases} \delta_0 & \text{if } t < 2015 \\ 1 - (1 - \delta_0)(1 - \delta_1 I_0(t) G) & \text{if } t \geq 2015 \end{cases} \tag{1}$$

where $I_0(t)$ is a function indicating whether the new interventions have been introduced by year t (1 if the interventions have been introduced, 0 otherwise), and G is the fraction of HIV-diagnosed individuals who are covered by the intervention once it is introduced. Due to the uncertainty regarding these new interventions, we assign three separate prior distributions: one to represent the uncertainty regarding the timing of the new interventions, one to represent the uncertainty regarding the coverage (G) and one to represent the uncertainty regarding the δ_1 parameter. The prior for the time to the introduction of the new interventions

(in years after 2015) is the same as that for other new interventions: a Weibull distribution with a median of 10 years and a shape parameter of 0.55.

1.1.6 Coverage of intensified risk reduction counselling (of diagnosed HIV-positives)

The prior for the coverage parameter is uniform on the interval (0, 1), reflecting the uncertainty regarding the level of coverage that would be possible.

1.1.7 Reduction in unprotected sex following intensified counselling in HIV-positive adults

The prior distribution on the δ_1 parameter is a beta distribution with a mean of 0.5 and a standard deviation of 0.22; the mean corresponds to the average of the percentage reductions in unprotected sex in the three previously-described South African trials [19-21], and the standard deviation was chosen so that the upper 97.5 percentile would correspond to the 90% reduction observed in the most promising of these three trials.

1.2 Antiretroviral treatment (ART)

The model of adult ART initiation and survival is described elsewhere [23]. Briefly, ART initiation is assumed to occur either at the time of HIV diagnosis (in a specified fraction of cases) or at a later date (depending on some specified delay between diagnosis and subsequent ART initiation if the individual is ART-eligible). Mortality after ART initiation is assumed to depend on the individual's baseline CD4 count, age, sex and time since ART initiation.

1.2.1 Fraction of patients starting ART with CD4 <200 who are virally suppressed

The model has been extended to include assumptions about viral load distributions and HIV infectiousness as a function of viral load, for the purpose of calculating average levels of infectiousness after ART initiation. Suppose that random variable $X_{a,s}$ is the difference between the maximum viral load and the actual viral load, on the logarithmic scale, in individuals with ART status a (0 = untreated, 1 = treated) and CD4 stage s (in untreated individuals, s refers to the current CD4 stage, while in treated individuals s refers to the CD4 stage at the time of ART initiation). Stage 1 corresponds to acute HIV infection, while stages 2-5 correspond to CD4 counts of ≥500, 350-499, 200-349 and <200. The maximum viral load is set to 6 on the $\log_{10}$ scale (although higher values are possible, these have little effect on the HIV transmission dynamics in which we are interested). Variable $X_{a,s}$ is assumed to be Weibull-distributed, with parameters $\omega_{a,s}$ and ϕ. The probability of viral suppression (a viral load of less than 400 copies/ml) in treated individuals is thus

$$\exp\left(-\omega_{1,s}(6-\log 400)^{\phi}\right), \tag{2}$$

from which it follows that if $V_s(t)$ is the probability of viral suppression in year t,

$$\omega_{1,s} = \frac{-\ln(V_s(t))}{(6-\log 400)^{\phi}}. \tag{3}$$

In fitting Weibull distributions to viral load data from both treated [24, 25] and ART-naïve South Africans [26], we have found that a ϕ parameter of 1.5 produces reasonable fits. In the period up to mid-2013, the $V_5(t)$ parameter (representing the rate of viral suppression in patients starting ART with CD4 <200 cells/µl) has been set to 0.77, based on data from South Africa's public sector ART programme [27] (a similar estimate of 0.75 was estimated for 2012 in a recent analysis of data from the National Health Laboratory Service [28]). Substituting $V_s(t) = 0.77$ into equation (3) yields a $\omega_{1,5}$ estimate of 0.042. Based on fitting the Weibull model to the median and inter-quartile range of viral loads prior to ART initiation in South Africans who almost all had CD4 counts of <200 cells/µl [26], we estimate the $\omega_{0,5}$ parameter to be 0.635.

We assume that if x is the difference between the maximum viral load and the actual viral load (on the logarithmic scale), the HIV transmission risk per act of sex is

$$c\exp\left(-\theta x^{\phi}\right), \tag{4}$$

where c is the maximum HIV transmission risk (when $x = 0$) and parameter θ determines the extent of the association between viral load and HIV transmission risk. Including $\phi > 1$ in the above equation ensures that the effect of viral load is less substantial at higher viral load levels than at lower viral load levels [29]. For reasons of mathematical convenience, explained below, we use the same value of $\phi = 1.5$ as estimated in the model of viral load distributions. The θ parameter is estimated by noting that if the factor by which infectiousness increases, per unit increase in viral load, is of the order of 2.5 [30-32], this implies that

$$\frac{-\frac{d}{dx}\left[c\exp\left(-\theta x^{\phi}\right)\right]}{c\exp\left(-\theta x^{\phi}\right)} = \ln(2.5). \tag{5}$$

From this it follows that $\theta\phi x^{\phi-1} = \ln(2.5)$. Substituting $\phi = 1.5$ and $x = 2$ [30, 31] yields $\theta = 0.432$. The average HIV transmission probability, for patients with ART status a and CD4 stage s, is then

$$\int_0^{\infty} \omega_{a,s}\phi x^{\phi-1}\exp\left(-\omega_{a,s}x^{\phi}\right)c\exp\left(-\theta x^{\phi}\right)dx = c\int_0^{\infty} \omega_{a,s}\phi x^{\phi-1}\exp\left(-\left(\theta+\omega_{a,s}\right)x^{\phi}\right)dx$$
$$= \frac{c\omega_{a,s}}{\omega_{a,s}+\theta}. \tag{6}$$

The advantage of using the same value of ϕ = 1.5 in the modelled relationship between viral load and HIV transmission risk is thus that it ensures a simple mathematical expression for the average probability of HIV transmission. From equation (6), the ratio of the infectiousness after ART initiation to that prior to ART initiation is

$$R_s = \frac{\omega_{1,s}}{\omega_{1,s} + \theta} \Big/ \frac{\omega_{0,s}}{\omega_{0,s} + \theta}. \qquad (7)$$

Substituting the values of $\omega_{1,5}$ = 0.042 and $\omega_{0,5}$ = 0.635 into this equation yields an R_5 estimate of 0.149. This is somewhat higher than the relative risk estimates of 0.04-0.08 estimated from randomized controlled trials [33, 34], but lower than the relative risk of 0.36 estimated in a recent meta-analysis of observational studies [35]. For patients who start ART at higher CD4 counts, data show that although they have lower baseline viral loads [36], they also have lower rates of virological failure after ART initiation [37], which suggests similar relative reductions in infectiousness across baseline CD4 categories. It is therefore assumed that the relative reduction in infectiousness is the same in all patients starting ART (i.e. R_s = R_5 for s < 5). Rates of viral suppression in patients who start ART at CD4 counts >200 cells/μl are calculated from equation (7), assuming that average viral load levels in untreated patients decrease by 0.18 for each 100-cell increase in the CD4 cell count [36] (which determines the $\omega_{0,s}$ values).

The model allows for uncertainty in future rates of viral suppression. Although a 77% rate of viral suppression has been assumed as the baseline, an 86% rate of viral suppression has been measured at 12 and 24 months after ART initiation in South African programmes participating in the IeDEA Southern Africa collaboration [38]. This higher rate of viral suppression probably reflects better access to resources in IeDEA programmes. A recent systematic review of ART adherence interventions in Africa estimates that SMS reminders and intensified adherence counselling in combination with treatment supporters could increase the odds of viral suppression by 1.55 (95% CI: 1.01-2.38) and 1.46 (95% CI: 1.09-1.97) respectively [39]. If such interventions were introduced in South Africa and an odds ratio of 2 were assumed (towards the upper end of the quoted 95% confidence intervals), this would yield an increase in viral suppression from 77% to 87%. Recent data from KwaZulu-Natal, collected following the introduction of adherence interventions, suggest viral suppression rates of between 85% and 90% in people who reported being on ART [25]. Another approach to defining a 'best case scenario' is to consider the 0.04 relative rate of transmission observed in the HPTN052 trial, when comparing individuals who started ART early to those in whom ART was deferred [33]. Substituting R_5 = 0.04 and $\omega_{0,5}$ = 0.635 into equation (25) yields an $\omega_{1,5}$ estimate of 0.011, which is equivalent to a virological suppression rate of 94% (from equation (21)).

Although these interventions suggest potential improvements in viral suppression in future, there are also a number of factors that may contribute to deteriorating viral suppression. There is particular concern that drug stockouts are becoming more frequent as the South African ART programme expands [40]. Increasing drug resistance could lead to declines in viral suppression; for example, Phillips *et al* [41] predict based on mathematical modelling that if levels of primary drug resistance in patients starting ART are around 5-10% (a level that is probably plausible for South Africa [42]), the rate of viral suppression one year after ART initiation will decline from 77% to 63% over 15 years, in the absence of any changes to

drug regimens and patient monitoring. Data from a South African community suggest a viral suppression rate of only 66% in individuals who reported that they were receiving ART [24], possibly due to low levels of adherence in this community. With this baseline, Phillips *et al* predict that a long-term viral suppression rate of 52% would be more likely [41], which we consider a 'worst case' scenario. To represent the uncertainty regarding future viral suppression, we assign a beta prior to the ultimate rate of viral suppression that applies in 2017 and future years. This beta prior has a mean of 0.77 and a standard deviation of 0.11 (with 2.5 and 97.5 percentiles of 0.52 and 0.94 respectively). The mean of 0.77 corresponds to the baseline assumption for 2012/13 [27], and the standard deviation has been chosen so that the upper and lower limits of the 95% confidence interval correspond to the best- and worst-case scenarios respectively.

1.2.2 Time to the introduction of universal ART eligibility

It is unclear if and when South African ART eligibility criteria will be extended to include all HIV-positive individuals (regardless of CD4 count, pregnancy or clinical stage). The START and TEMPRANO trials have both found that immediate ART initiation leads to better health outcomes than ART deferred according to previous guidelines [43, 44], and based on this the WHO will soon be issuing new guidelines recommending universal ART eligibility. It is likely that South African ART eligibility criteria will change to be consistent with WHO guidelines, although it is possible that local eligibility will not change, particularly as there are concerns about the fiscal implications of further expansion of eligibility criteria. We adopt the same approach as before to represent the uncertainty, assigning a Weibull distribution to represent the uncertainty regarding the timing of the change in eligibility criteria (in years after 2015), with a median of 10 years and a shape parameter of 0.55.

1.2.3 Time to the introduction of point-of-care (POC) CD4 testing

In a review of African studies that have examined linkages between HIV diagnostic services and ART services [45], the median proportion of South African patients who received CD4 testing following HIV diagnosis was around 75% and the median proportion of those receiving CD4 testing who collected their test results was around 80%. Of those who were determined to be ART-eligible, the average proportion who started ART was around 67%. This suggests that of all individuals who are newly diagnosed and ART-eligible, the proportion who actually start ART within a few months of diagnosis is only about 40% (0.75 × 0.80 × 0.67). We have therefore set the rate of linkage to care in 2012/13 to 0.4, for all individuals who are ART-eligible, asymptomatic and not pregnant.

The rate at which newly-diagnosed adults link to ART could change in future, for two reasons. Firstly, the introduction of point-of-care (POC) CD4 testing would mean that patients could have their CD4 count determined at the same time that HIV diagnosis occurs, and this would reduce drop-out. For example, Larson *et al* [46] found that the introduction of POC CD4 testing led to an increase in linkage to care following HIV diagnosis in South Africa (RR 1.25, 95% CI: 1.00-1.57), and Govindasamy *et al* [47] found that the proportion of CD4-tested individuals who received their test results increased from 73% to 98% (RR 1.34) following the introduction of POC testing. This suggests a theoretical increase from

40% to 52% if a RR of 1.3 is assumed (mid-way between the RR values of the two studies). Secondly, the switch to universal ART eligibility would obviate the need for baseline CD4 testing. This could theoretically increase the proportion of newly-diagnosed individuals starting ART from 40% to 67% (removing the factors of 0.75 and 0.8 from the calculation in the previous paragraph). In the period after 2015, we model the rate of linkage to care in asymptomatic, non-pregnant adults as

$$0.4\times(1-I_2(t))(1-I_3(t))+(I_2(t)+I_3(t)-I_2(t)I_3(t))l_0^M \tag{8}$$

where $I_2(t)$ is an indicator of whether POC CD4 testing has been introduced up to year t (1 if so, 0 otherwise); $I_3(t)$ is similarly an indicator of whether universal ART eligibility has been introduced; and l_0^M is the proportion starting ART immediately after diagnosis, after the introduction of POC CD4 testing or universal ART eligibility.

Uncertainty regarding the time to the adoption of POC CD4 testing (in years after 2015) is modelled in the same way as other new interventions, using a Weibull distribution with a median of 10 years. This determines in which years $I_2(t) = 1$.

1.2.4 Fraction starting ART immediately after diagnosis if POC CD4/universal ART available

Based on the South African evidence reviewed in section 1.2.3, we model the uncertainty regarding the l_0^M parameter by assigning to this parameter a beta distribution with a mean of 60% and a standard deviation of 10%. The mean of this distribution is midway between the 52% and 67% estimates, and the 2.5 and 97.5 percentiles of the distribution are at 40% and 79% respectively. The standard deviation has thus been chosen so that in a worst case scenario the interventions would lead to no improvement in linkage to ART, while in a best case scenario the interventions would increase linkage to care to levels similar to those assumed in pregnant women and OI patients (80%).

1.2.5 Mean time to women starting ART if ART is not started at time of diagnosis

In the period up to mid-2014, the modelled rates of ART initiation in previously-diagnosed adults are calculated from reported numbers of adults starting ART in each period (after subtracting the number of adults who are estimated to have started ART soon after diagnosis). The assumed rate of ART initiation in the CD4 200-349 category is assumed to be 0.7 times that in the CD4 <200 category, while the corresponding multipliers in the CD4 350-499 and ≥500 categories are 0.5 and 0.4 respectively, assuming individuals are diagnosed and ART-eligible. (These assumptions are based primarily on the observed relative rates of ART initiation in ART-eligible individuals in different CD4 categories [48], and are consistent with the relative rates at which individuals enrolled in pre-ART care return for regular CD4 testing [49, 50].) Table S1.1 shows the assumed absolute numbers starting ART in each period and the resulting model estimates of ART initiation rates in previously-diagnosed adults (expressed as average treatment delays).

Table S1.1: Assumed annual numbers of patients starting ART in South Africa, and implied average treatment delays in previously-diagnosed patients with CD4 counts <200 cells/µl

	Men (15+)	Women (15+)	Children (<15)	Implied ART delay	
				Men (15+)	Women (15+)
Pre-2000	0	0	0	-	-
2000-01	2 808	3 553	464	217.4	238.1
2001-02	4 476	5 664	740	181.8	208.3
2002-03	5 340	6 757	883	196.1	238.1
2003-04	7 953	11 442	2 246	161.3	181.8
2004-05	22 852	44 318	6 595	68.0	54.9
2005-06	43 258	84 447	13 730	52.6	37.3
2006-07	54 579	104 965	15 076	62.9	41.3
2007-08	76 045	147 733	19 341	54.1	32.9
2008-09	115 407	216 722	30 972	32.9	20.3
2009-10	158 492	285 854	34 754	22.4	13.9
2010-11	185 562	355 024	43 143	19.4	10.4
2011-12	180 271	353 763	36 676	32.5	16.7
2012-13	185 843	342 701	28 999	40.0	17.4
2013-14	155 801	283 732	23 547	50.0	19.6

Because we do not yet have data on the absolute numbers starting ART after mid-2014, we specify the treatment delay parameters directly for this period. Our baseline simulations suggest that in women with CD4 counts of <200 cells/µl, this average delay was around 10 months in 2010/11 but gradually increased to just under 20 months in the more recent periods, possibly as a result of ‘crowding out’ of sicker patients as ART eligibility criteria have expanded to include healthier patients. However, these results should be interpreted with caution, as the estimates are sensitive to assumptions about linkage to care after diagnosis, which are difficult to determine precisely. In the period since 2009, the delay in men was on average around 2 times that in women (the ratio was substantially lower in the early 2000s, when men had better access to ART through private healthcare). Mujugira *et al* [48] found that among East African adults who were previously diagnosed and were subsequently determined to have CD4 counts <200 cells/µl, the average delay to ART initiation was around 6 months (with no significant difference between men and women). However, this is likely to be a lower bound on the delay that would apply in practice, (a) because the study was conducted in the context of a randomized trial, in which patients were intensively counselled and monitored; and (b) because most of those with CD4 counts <200 cells/µl had only recently become eligible for ART, so the cohort included relatively few individuals who were diagnosed in previous periods, who might have chosen to delay ART until they were at a more advanced stage of disease. It is likely that delays would be longer in field settings, where access to services may be limited. There is concern that South Africa’s public sector ART programme may be over-stretched and that limited future availability of resources might effectively lead to restricted ART access, even if the guidelines officially endorse early ART initiation [51]. To represent the uncertainty regarding likely future delays, we assign a gamma prior distribution to the mean time to ART initiation in women with CD4 <200 cells/µl who have not previously started ART. This prior distribution has a mean of 18 months and a standard deviation of 8 months (with 2.5 and 97.5 percentiles at 5.9 and 36.7 months respectively). The mean thus corresponds to the baseline estimates in 2012-13 and 2013-14, and the standard deviation has been chosen so that in a best case scenario rates of

ART initiation would be similar to those in the East African trial. The upper limit of 36.7 months corresponds to a worst case scenario in which treatment delays return to those seen in 2005/6, when the public sector ART programme was still in its early stages. The average delay in men is assumed to be 2 times that in women, consistent with the model estimates over the period from mid-2009 to mid-2014.

1.3 Behaviour change parameters

The model of sexual behaviour is described in detail elsewhere [23]. Briefly, individuals are stratified into high and low risk groups (and within the female high risk group there is further stratification according to whether women are sex workers). Within each risk group, individuals are further stratified according to whether they are married/cohabiting or unmarried and not cohabiting with any current partners. Rates of partnership formation and entry into marriage are assumed to depend on age, sex and risk group. Rates at which men visit sex workers are also assumed to depend on age, sex and marital status.

1.3.1 Average partner age differences in non-marital relationships

For women who are aged x and in non-marital relationships, the age distribution of non-marital partners is assumed to have a mean of (x + 3), and a standard deviation of 3 years, consistent with partner age distributions reported by young women in various South African studies [52-56]. This distribution is adjusted to take into account the actual number of men available at each age.

Although the model assumes that age preferences in non-marital relationships have been stable over time, recent surveys suggest that age differences may have increased at young ages: the proportion of young women who report a partner age difference of 5 years or more has been estimated at 34-40% in recent surveys [57-59], in contrast to the level of 18.9% estimated in the model based on earlier data [52-56]. However, at the same time there have been behaviour change programmes to discourage young women from having relationships with older men. For example, the "Sugar daddies destroy lives" campaign was launched in KwaZulu-Natal in 2012, and a study in this province has found evidence of significant reductions in partner age differences reported by young women [60]. Cluver *et al* [61] have also found that adolescent South African girls in households that were receiving child support grants (CSGs) were 71% less likely to report having older partners (>5 year age difference) than girls in households that are not receiving CSGs. A trial of a cash transfer intervention in Malawi has also shown that providing cash payments to young women reduces their likelihood of reporting a partner over the age of 25 by 79% [62]. This suggests that both the recent increases in access to the CSG (with the age of eligibility increasing from 16 to 18 in 2011) and the potential future introduction of economic empowerment/cash transfer programmes for young women could lead to reductions in average partner age differences.

To represent the uncertainty regarding changes in age-disparate relationships, we assign a gamma prior distribution to represent the range of possible future average partner age differences in short-term relationships, with this distribution having a mean of 3 years (the same as in the baseline scenario) and a standard deviation of 1 year. This allows for the

possibility that mean partner age differences could either increase (in line with the trends observed in recent surveys) or decrease (in response to interventions). The standard deviation of the distribution of partner age differences is assumed to increase or decrease in proportion to the mean. The 2.5 and 97.5 percentiles of the distribution of mean partner ages are at 1.37 years and 5.25 years respectively. With a mean and standard deviation of 1.37 years, the probability of a more than 5 year age difference would be 2.6% (86% lower than the baseline, and thus consistent with the reductions that we might optimistically expect if cash transfer interventions were introduced and had effectiveness similar to the previously-cited studies). With a mean and standard deviation of 5.25 years, the probability of a more than 5 year age difference would be 38.6% (consistent with the levels of 34-40% reported in recent surveys).

1.3.2 Ratio of marriage rates in 2016/17 to those in 2011/12

Although rates of marriage in South Africa have been steadily declining over the last few decades [63-66], the model assumes that age-specific marriage rates have been constant up to 2011/12. A number of factors have been suggested as reasons for the low and declining rates of marriage in South Africa, including the rising cost of *lobolo* (bride wealth), increasing employment opportunities for women, and the historic effects of apartheid legislation, which prevented African couples from settling permanently in 'white' urban areas [63, 64]. It is possible that interventions might reverse the downward trend in marriage rates, for example, increasing access to finance for men who cannot afford *lobolo*, increasing employment opportunities in rural communities (which would lead to less labour migration) and promoting marriage through behaviour change communication programmes. However, there have been no studies to evaluate the effect of such interventions. In the light of this uncertainty, we adopt the same approach as used to model uncertainty regarding changes in sexual debut: in the period after 2012, we apply an adjustment factor to the rates of marriage that applied in 2011/12, and to represent the uncertainty around this adjustment factor, we assign a gamma prior distribution with a mean of 1 and a standard deviation of 0.2.

1.3.3 Ratio of sexual debut rates in 2016/17 to those in 2011/12

Rates of sexual debut are assumed to have remained constant up to 2012, given inconsistent evidence of trends from different studies [57, 58, 67, 68]. However, we consider possible changes to rates of sexual debut after 2012 in the uncertainty analysis. It is possible that interventions and behaviour change communication programmes may lead to later sexual debut, as has been seen in Uganda, where 27-36% reductions in rates of youth sexual experience were reported [69]. A cash transfer intervention in Malawi was also associated with a 36% reduction in female rates of sexual debut, although this was not statistically significant [62]. A similar trial in Kenya, which provided support to cover adolescent school costs, also found a 43% reduction in sexual debut that was nearly statistically significant [70]. However, it is also possible that rates of sexual debut may be increasing, in line with the recent HSRC survey results, which found that the proportion of young men who reported sexual debut prior to age 15 increased by 48% between 2008 and 2012 [57]. In the period after 2012, we apply an adjustment factor to the rates of sexual debut that applied in 2011/12, to represent the effect of possible changes in rates of sexual debut. To represent the uncertainty around this adjustment factor, we assign a gamma prior distribution with a mean

of 1 and a standard deviation of 0.2. The 2.5 and 97.5 percentiles correspond to a 35% reduction and a 43% increase in sexual debut rates respectively, so that the lower limit corresponds to the reduction that we might optimistically expect if cash transfer interventions/awareness programmes were fully rolled out and were effective, and the upper bound corresponds to a scenario in which the recent HSRC survey results represent a true increase in sexual debut in recent years.

1.3.4 Odds of condom use in marital relationships (relative to 1998)

Rates of condom use are assumed to depend on age, sex, type of relationship and knowledge of HIV-positive status. Rates of condom usage are also assumed to change over time; this time-dependency represents the effect of HIV communication programmes and condom promotion campaigns, which were introduced in the 1990s and early 2000s, but which have since seen a decline in funding [71]. The parameter $\gamma_{2,l}(x,t)$ represents the probability that an HIV-negative woman aged *x* uses a condom in an act of sex with a partner of type *l* at time *t* (time is measured in years since 1985). This parameter is calculated in relation to an arbitrary 'baseline' rate of condom usage, γ^*, which is the probability of condom use for a woman aged 20 in a short-term relationship in 1998. The following formula is used to calculate $\gamma_{2,l}(x,t)$:

$$\ln\left(\frac{\gamma_{2,l}(x,t)}{1-\gamma_{2,l}(x,t)}\right) = \ln\left(\frac{\gamma^*}{1-\gamma^*}\right) + \chi_l + \nu_l(x-20) + \varsigma_l(t) \qquad (9)$$

where

$\exp(\chi_l)$ = the odds of using a condom in relationship type *l*, relative to that in short-term relationships (l = 0), in 1998;

$\exp(\nu_l)$ = the factor by which the odds of condom use reduces, per year of age;

$\exp(\varsigma_l(t))$ = the odds of using a condom in year *t*, relative to that in 1998, for relationship type *l*.

In the period up to 2011, the $\varsigma_l(t)$ function is a linear combination of a constant term and two cumulative Weibull distribution functions. The constant term represents the initial rate of condom usage, prior to the start of the HIV epidemic in South Africa, the first Weibull distribution corresponds to the increase in condom usage following the introduction of HIV communication programmes in the mid-1990s, and the second Weibull distribution represents the reversal in condom usage rates in recent years. Due to the uncertainty regarding condom usage trends after 2012 (discussed below), a different formula is used to model patterns of condom usage after 2012. In mathematical terms,

$$\varsigma_l(t) = \begin{cases} \kappa_l^1 + \left(\kappa_l^2 - \kappa_l^1\right)\left(1 - 0.5^{\left(t/M_l^1\right)^{Q_l}}\right) - \left(\kappa_l^2 - \kappa_l^3\right)\left(1 - 0.5^{\left(t/M_l^2\right)^{2Q_l}}\right) & \text{if } t \le 26 \\ \varsigma_l(26)\left(31 - t\right)/5 + \kappa_l^4\left(t - 26\right)/5 & \text{if } 26 < t \le 30 \\ \kappa_l^4 & \text{if } t > 30 \end{cases} \quad (10)$$

where t is time in years since 1985, and the other variables are defined as follows:
κ_l^1 represents the initial rate of condom use in relationship type l, in 1985 (relative to the baseline in 1998);
$\kappa_l^2 - \kappa_l^1$ represents the increase in condom use in relationship type l, following initial HIV communication programmes;
$\kappa_l^2 - \kappa_l^3$ represents the reduction in condom use in relationship type l, following reductions in condom promotion/risk compensation;
κ_l^4 represents the ultimate rate of condom use in relationship type l, after 2015;
M_l^1 = the median for the first Weibull distribution;
M_l^2 = the median for the second Weibull distribution;
Q_l = the Weibull shape parameter controlling the speed of behaviour change in relationships of type l.

In calibrating the model to historic HIV prevalence data and condom usage data, the $\exp(\varsigma_1(26))$ parameter for marital relationships has been set at 1.78. There is much uncertainty regarding potential future trends in condom usage. In a worst case scenario, condom usage may continue to decline in future. There have already been significant cuts in funding for HIV communication programmes [71], and future budget constraints could lead to further reductions in funding for these programmes. Even if there are no reductions in funding, it is possible that rates of condom usage may decline due to reduced fear of HIV in the era of highly effective and easily accessible treatment – so-called 'risk compensation' or 'treatment optimism'. Studies from other African countries have documented increases in risk behaviour in the general population (or HIV-negative population) associated with ART optimism and ART rollout [72-74], and it is thus possible that similar changes in behaviour may be occurring in South Africa.

On the other hand, there are several ways in which levels of condom usage could be increased. Previous South African studies have shown that levels of condom usage are strongly associated with levels of exposure to HIV communication programmes [58, 75] as well as lifeskills programmes in schools [76], and renewed emphasis on condoms through HIV communication programmes and lifeskills programmes could therefore lead to greater condom use. In the most recent national HIV communication survey [58], the median level of reported condom use at last sex was 50%, while the level of condom use in the 10% of respondents with the highest level of exposure to HIV communication programmes was 63%; this suggests that the odds of condom usage could be increased by a factor of 1.7 if levels of exposure to HIV communication programmes were increased to those currently achieved in the top decile.

To represent the uncertainty regarding future trends in condom usage, we assign gamma prior distributions to the $\exp\left(\kappa_l^4\right)$ parameter. For each relationship type, the gamma mean is set to the $\exp\left(\varsigma_l(26)\right)$ parameter, and the standard deviation is set in such a way that the 2.5 percentile of the gamma distribution corresponds to 1. This means that on average condom usage is projected to remain stable after 2011, but in the most pessimistic scenarios, condom usage would be unlikely to fall below the levels in 1998 (when HIV communication programmes and condom distribution were still at relatively low levels). The 97.5 percentiles of the distributions for marital and non-marital relationships are 2.79 and 6.47 respectively (1.57 and 2.06 times the corresponding baseline values, and thus roughly consistent with the odds ratio of 1.7 that might optimistically be assumed if high levels of exposure to HIV communication programmes were achieved).

1.3.5 Odds of condom use in non-marital relationships (relative to 1998)

In calibrating the model to historic HIV prevalence data and condom usage data, the $\exp\left(\varsigma_1(26)\right)$ parameter for non-marital relationships has been set at 3.14. As explained in the previous section, there is significant uncertainty regarding likely future trends in condom usage, and the same factors that influence the rates of condom use in marital relationships are likely to influence condom use in non-marital relationships. As explained in the previous section, the gamma prior for the $\exp\left(\kappa_0^4\right)$ parameter has been assigned a mean of 3.14 and a standard deviation of 1.42, which yields 2.5 and 97.5 percentiles of 1.00 and 6.47 respectively.

1.3.6 Odds of condom use in SW-client relationships (relative to 1998)

In addition to the need for general HIV communication programmes, there may also be a need for more targeted approaches to promote condom usage in specific high risk groups. For example, a recent meta-analysis estimated that community empowerment initiatives in sex workers increase the odds of condom usage among sex workers and their clients by a factor of 3.27 (95% CI: 2.32-4.62) [77]. Although 94% of South African female sex workers reported using condoms with their last client in a recent national survey [78], non-use of condoms is significantly associated with being drunk (OR 2.6, 95% CI: 1.7-3.8). This suggests that substance abuse programmes for sex workers may be particularly important in increasing their consistency of condom use. Mathematical modelling also suggests that decriminalization of commercial sex could lead to substantial reductions in HIV incidence through its effect on sex workers' ability to negotiate consistent condom use with their clients [79].

In calibrating the model to historic HIV prevalence data and condom usage data, the $\exp\left(\varsigma_2(26)\right)$ parameter for sex worker-client relationships has been set at 3.80. The gamma prior for the $\exp\left(\kappa_2^4\right)$ parameter has been assigned the same mean (3.80) and a standard deviation of 1.42, which yields 2.5 and 97.5 percentiles of 1.00 and 8.44 respectively. The 97.5 percentile of 8.44 is 2.2 times the baseline value, and thus roughly consistent with the

value that would be expected if it were optimistically assumed that community empowerment efforts were rolled out and reached 50% of sex workers (0.5 + 0.5 × 3.27 = 2.14).

1.4 Prevention of mother-to-child transmission (PMTCT)

The modelling of mother-to-child transmission has been described previously [23, 80]. In summary, children can acquire HIV from their mothers either perinatally (at/before birth) or postnatally (through breastmilk). The perinatal transmission rate depends on the fraction of HIV-positive mothers who are diagnosed positive during pregnancy and the rate at which they start long-term ART or short-course antiretroviral prophylaxis. The postnatal transmission rate depends on the fraction of breastfeeding mothers who are receiving ART as well as the rate at which mothers seroconvert while breastfeeding (since mothers who are in the acute stage of HIV infection while breastfeeding are assumed to have a high postnatal transmission risk). The postnatal transmission risk also depends on the type of breastfeeding, with most HIV-diagnosed mothers being assumed to progress from an initial phase of exclusive breastfeeding (EBF), when the transmission risk is relatively low, to a period of mixed feeding, in which there is assumed to be an increased transmission risk.

1.4.1 Rate of retesting in late pregnancy

High rates of antenatal HIV testing have been achieved in South Africa, and it is assumed that the fraction of mothers tested for HIV antenatally reached 98% in 2011/12 [81] and will remain at 98% in future. In addition, the model makes allowance for retesting in late pregnancy, which is potentially important in identifying women who experience seroconversion after their first HIV test. In the period up to 2006, there is assumed to have been no retesting prior to delivery of mothers HIV-negative at their first antenatal visit. Recent studies suggest that the proportion of women testing negative who get tested again in late pregnancy has been steadily increasing over time [82, 83], with the most recent data suggesting a retesting frequency of 46% in 2011 [83]. In a best case scenario, this proportion may rise to 100%, but in a more conservative scenario, this proportion might change relatively little from the rate of 45% assumed for the 2011/2012 period. We assign a beta prior distribution to represent the range of uncertainty around the proportion of women who are offered retesting in late pregnancy, with this beta prior having a mean of 75% and a standard deviation of 12% (2.5. and 97.5 percentiles at 48% and 94% respectively). Women who are diagnosed HIV-positive following retesting are assumed to be as likely to receive short-course antiretroviral prophylaxis and long-term ART as women who are diagnosed at their first antenatal visit.

1.4.2 Fraction of newly-diagnosed pregnant women who start ART prior to delivery

In 2011/12, national statistics show the proportion of newly-diagnosed pregnant women initiating ART rose to 75.4% in [84]. It is likely that this proportion increased in subsequent periods, following the introduction of WHO option B at the start of 2013, which eliminated the need for CD4 testing prior to ART initiation and thus simplified the ART initiation

process. The Department of Health target is to ultimately increase the proportion of HIV-positive mothers initiating ART during pregnancy to 100% [2], but this may be unrealistic given the challenges associated with ART initiation in pregnancy, and given that some women do not receive any antenatal care prior to delivery. Linkage rates as high as 95% have been reported [85], and these might be considered a best case scenario. To represent the uncertainty regarding the proportion of women who start ART prior to delivery, following an HIV diagnosis in 2016 ($l_2(s, 2016)$), we assign a beta prior with a mean of 90% and a standard deviation of 4% (2.5 and 97.5 percentiles of 81% and 96% respectively). The mean and standard deviation have thus been chosen so that the upper limit on the confidence interval corresponds to the best case scenario, while the lower limit reflects negligible improvement relative to the baseline.

1.4.3 Proportionate increase in mean duration of ART prior to delivery

The model of mother-to-child transmission has been extended to allow the perinatal transmission probability to depend on the average duration of ART prior to delivery. For women who start ART during pregnancy, in CD4 stage s, the probability of perinatal transmission is assumed to be of the form

$$a + b_s R^x, \tag{11}$$

where a is the minimum transmission risk (the risk that might be expected in women who started ART prior to conception), b_s is the difference between the maximum and minimum transmission risk (the maximum being that which applies if ART is initiated just prior to delivery), R is the factor by which the difference reduces per week of ART prior to delivery, and x is the number of weeks of ART received prior to delivery. If $g(x)$ is the probability density function describing the distribution of ART durations in the baseline scenario (before any interventions to improve ART initiation during pregnancy), and this density is assumed to be of gamma form, then the average probability of perinatal transmission in the baseline scenario is

$$\begin{aligned}\int_0^\infty g(x)\left(a + b_s R^x\right) dx &= a + b_s \int_0^\infty \frac{\lambda(t)^\alpha x^{\alpha-1} \exp\left(-\lambda(t)x\right)}{\Gamma(\alpha)} R^x dx \\ &= a + b_s \left(\frac{\lambda(t)}{\lambda(t) - \ln(R)}\right)^\alpha . \end{aligned} \tag{12}$$

where α and $\lambda(t)$ are the parameters of the gamma distribution. Based on South African data sources [86-89], the mean and standard deviation of the gamma distribution in the baseline scenario have been set to 10.6 weeks and 8 weeks respectively (α = 1.7556 and $\lambda(t)$ = 0.1656 for t < 2010), and the R parameter has been set to 0.9. Parameter a has been set to 0.006, the average transmission risk from studies that evaluated the perinatal transmission rate from mothers who started ART prior to conception [86, 88, 90] (Table S1.14).

The remaining b_s parameter is estimated by equating expression (12) to the known average perinatal transmission probability that existed in the baseline scenario. This is calculated separately for women who started ART during pregnancy with CD4 <200 (s = 5) and women

who started ART in pregnancy at higher CD4 counts (s < 5); based on previous research these average transmission probabilities are assumed to be 0.031 and 0.015 respectively [86-88, 91-96]. The resulting estimates of the b_s parameter are 0.059 and 0.021 respectively.

It is likely that there has already been some improvement in the average duration of ART, relative to the baseline scenario. The South African 2010 PMTCT guidelines recommended integration of ART provision into PMTCT services [97], which led to more rapid initiation of ART during pregnancy. For example, Van Schalkwyk *et al* [89] found that the median duration of ART prior to delivery increased from 7.7 weeks in the 2008-9 period to 13.1 weeks in 2010 following the introduction of the new guidelines. A similar median of around 12 weeks has been observed in the period following 2010 in the Eastern Cape, and even higher rates of ART uptake were measured from 2012 [98]. Stinson *et al* [99] documented a more substantial difference (about 7 weeks) in the median time to ART initiation when comparing the ART referral model to the integrated ART model. There have also been steady improvements over time in the mean gestational age at first antenatal booking; for example, the Department of Health [2] reports that the proportion of mothers who had their first antenatal visit before 20 weeks gestation has increased from 37.5% in 2010/11 to 50.6% in 2013/14. It is therefore assumed that the mean duration of ART increased by 25% in 2010-11 and by 50% in 2011-12 (relative to the mean duration in the pre-2010 period). This means setting $\lambda(t)$ = 0.1104 over the 2011-2012 period, which leads to a 22% reduction in the probability of perinatal transmission from mothers with initial CD4 counts <200 cells/µl. Following the introduction of WHO option B at the start of 2013, it is likely that the delay in ART initiation would have been reduced even further, since the removal of the CD4 restriction would have eliminated the delay associated with CD4 testing. In a best case scenario, we might expect a doubling of the mean duration of ART relative to the pre-2010 period, i.e. an average of 21 weeks of ART, which is what might be expected if the mean gestational age at the first antenatal visit was around 18 weeks and all HIV-positive women were to start ART immediately upon diagnosis. In a more conservative scenario we might expect no improvement over the 50% increase achieved in the 2011-2012 period. To represent the uncertainty surrounding the percentage increase in mean ART duration, we assign a gamma prior distribution with a mean of 70% and a standard deviation of 14% (2.5 and 97.5 percentiles of 45% and 100% respectively).

1.4.4 Relative rate of short-course ARV uptake if long-term ART not started prior to delivery

In the period from the end of 2012 to the end of 2014, WHO option B has been official policy in South Africa, and since the start of 2015, option B+ has been official policy. However, there has been a lack of clarity regarding the provision of short-course antiretroviral prophylaxis to women who – for whatever reason – do not start long-term ART during pregnancy. Western Cape guidelines recommend that pregnant HIV-positive women who are not able to start long-term ART immediately should receive AZT prophylaxis, and if an HIV-positive mother presents in labour but is not on ART, short-course ARV prophylaxis should be initiated [100]. However, recent national guidelines make no mention of short-course antiretroviral prophylaxis [101]. Recent data from Botswana show that the introduction of WHO Option B in Botswana has led to an *increase* in the fraction of HIV-positive women who receive no ARV prophylaxis during pregnancy [102], which suggests that women who

do not start long-term ART are less likely to be offered short-course ARV prophylaxis than they were previously. The simplification of PMTCT and the lack of clarity regarding the provision of short-course ARV prophylaxis probably mean that some antenatal clinics might *only* offer long-term ART, as has been the case in Malawi following the introduction of WHO Option B+. In addition, the mothers who do not start long-term ART despite being offered therapy are likely to be a select group, different from the mothers who qualified only for short-course ARV prophylaxis in the period up to 2012 (i.e. if they have refused long-term ART, they might be as likely to refuse short-course ARV prophylaxis). We have therefore assigned a uniform (0, 1) prior distribution to represent the uncertainty regarding the relative rate of short-course ARV uptake in the post-2012 period, in women who do not start long-term ART. The choice of a vague prior reflects the lack of data regarding the relative rate of short-course ARV uptake since the switch to WHO options B and B+.

1.4.5 Relative infectivity of HIV-positive women on long-term ART (breastfeeding)

In the period up to 2011/12, there is assumed to be an 80% reduction in postnatal transmission rates in women on ART, relative to breastfeeding mothers who are untreated. This is calculated as one less the ratio of the average monthly postnatal transmission risk in five studies (0.0018) [91-94, 103] to the average monthly transmission risk of 0.0097 for untreated mothers, estimated when a similar model was previously fitted to South African data [104]. However, the model of postnatal transmission is simplistic because it implicitly assumes that all mothers who initiate ART during pregnancy remain on ART throughout the breastfeeding period. There is concern that rates of retention in care may be poor in the post-partum period, particularly in the context of WHO option B+ [105, 106]. In a recent study conducted in Cape Town it was found that 28% of mothers who initiated ART during pregnancy dropped out of ART care during the 6 months after delivery [106]. If it were conservatively assumed that all of these women stopped ART for the entire duration of the breastfeeding period, the reduction in postnatal transmission would be only 80% × (1 – 0.28) = 58%. On the other hand, it is possible that the ART adherence interventions described in section 1.2.1 could contribute to improvements in viral suppression in future, and hence lead to further reductions in postnatal transmission rates. The effect of plasma viral load on perinatal and postnatal transmission rates [107] is similar to that on heterosexual transmission probabilities [30-32], and hence the 96% reduction in HIV transmission probabilities due to ART in the HPTN 052 trial [33] should – in theory – also be possible in the context of postnatal HIV transmission. A beta distribution is used to represent the uncertainty regarding the future reduction in the postnatal HIV transmission risk due to ART. This distribution has a mean of 0.8 (the same as the baseline value) and a standard deviation of 0.1 (2.5 and 97.5 percentiles at 57% and 95% respectively). The standard deviation has thus been chosen so that the upper and lower limits of the 95% confidence interval correspond to the best case and worst case scenarios respectively.

1.4.6 Median duration of exclusive breastfeeding

In the period up to 2011, the model assumes that HIV-diagnosed women who practised EBF did so for a median of 2 months (up to a maximum of 6 months), after which 30%

discontinued breastfeeding completely and the remainder practised mixed feeding (i.e. continued breastfeeding while introducing complementary feeds), for a median of 7 months [108-110].

The benefits of EBF have been increasingly emphasized following the Tshwane declaration [111], with guidelines recommending 6 months of EBF for all mothers (as well as continued mixed feeding after 6 months) and the phasing out of the free provision of formula milk for HIV-positive mothers. The proportion of HIV-diagnosed women who avoid breastfeeding is assumed to have declined from 56% in 2010/11 to 20% in 2013/14, in line with data from a series of national PMTCT surveys [112]. It is also likely that there has been some increase in the median duration of EBF over time, although there are currently no data to determine the extent of this change. We assign a gamma prior distribution to represent the uncertainty regarding the median duration of EBF from 2013, the year in which the impact of the change in infant feeding policy is assumed to reach its maximum. This gamma distribution has a mean of 4 months and a standard deviation of 1 month (2.5 and 97.5 percentiles at 2.29 and 6 months respectively, assuming EBF does not continue for more than 6 months). The mean and standard deviation have thus been chosen so that the lower bound on the confidence interval corresponds to a 'worst case' case scenario, in which there is little change relative to the baseline scenario, and the upper bound corresponds to a 'best case' scenario, in which the median duration of EBF is consistent with that recommended in national guidelines.

1.4.7 Fraction of mothers discontinuing EBF who stop breastfeeding completely

It is also assumed that the proportion of mothers who stop breastfeeding completely (as distinct from introducing solids) at the time of ceasing EBF is likely to have declined from the baseline of 30%. To represent the uncertainty regarding the likely magnitude of the decline, we assign a beta prior distribution to the fraction of mothers discontinuing EBF who stop breastfeeding completely. This distribution has a mean of 15% and a standard deviation of 6% (2.5. and 97.5 percentiles at 5% and 28% respectively). The lower bound of the 95% confidence interval thus corresponds to a 'best case' scenario in which 95% of women continue to breastfeed after introducing solids, while the upper bound corresponds to a 'worst case' scenario in which there is negligible improvement relative to baseline.

1.5 Medical male circumcision

The modelling of male circumcision has been described previously [23]. The rate at which men get circumcised is assumed to be composed of two parts: the 'background' rate of male circumcision that would be expected in the absence of any efforts to promote male circumcision as an HIV prevention strategy, and the rate of male circumcision due to medical male circumcision (MMC) campaigns. In modelling the former, a cumulative Weibull distribution is used to represent the age-related changes in the prevalence of male circumcision prior to 2008. In modelling the latter, the model relies on reported numbers of men circumcised through MMC campaigns, which was 331 668 in 2013/14 [3]. The uptake of MMC is distributed across the HIV-negative population in proportion to the annual probability of acquiring a short-term (non-marital) partner, i.e. assuming that the men who

are most at risk of HIV are most motivated to get medically circumcised. This means that in each year up to 2013/14, a probability of MMC uptake is calculated for men who are in short-term relationships, and this is scaled down in proportion to the annual probability of acquiring a short-term partner, for each uncircumcised male.

The model estimates the annual probability of MMC uptake in 2013/14, for men in short-term relationships, to be 0.15. The Department of Health target is to increase the number of MMC operations to 1 000 000 per annum from 2014/15 [2], which would be equivalent to a roughly 0.68 probability of MMC uptake in men in non-marital relationships in 2014/15. These increases in MMC operations could potentially be achieved through a number of novel MMC promotion methods. For example, MMC campaigns during school holidays may be particularly important in boosting the uptake of MMC at young ages [113], while payments to compensate for time off work may be particularly important in motivating older men to get circumcised [114]. There also appears to be a strong association between men's self-reported exposure to HIV communications programmes and their intentions to get circumcised [58], which suggests that HIV communication programmes may be important in generating demand for MMC. Promotion of MMC to men seeking VCT may also be effective [115].

However, there are a number of reasons why it may be overly optimistic to set the future annual probability of MMC uptake to 0.68. Most importantly, there is likely to be a degree of intervention saturation, which has not been taken into account in the target setting. In the face of declining numbers of uncircumcised men, the absolute numbers of MMC operations may well decline. This would be particularly likely if many of the men who are currently uncircumcised belong to ethnic groups such as the Xhosa, in which MMC is considered culturally unacceptable [116, 117], or if most of the remaining uncircumcised men are at low HIV risk and are unlikely to see much benefit in getting circumcised. In the 2012 National Communication Survey [58], the fraction of uncircumcised men who said that they definitely intended to get medically circumcised in the next 12 months was only 2-4% in the Eastern Cape and Western Cape, the two provinces with the highest proportions of Xhosa speakers. This might be considered a lower bound on the future rate of MMC uptake in men in non-marital relationships, if most of the remaining uncircumcised men were resistant to the idea of MMC for cultural reasons.

We assign a beta prior distribution to reflect the uncertainty regarding the ultimate annual probability of MMC uptake in men in non-marital partnerships. This beta prior has a mean of 0.30 and a standard deviation of 0.17, which gives 2.5 and 97.5 percentiles at 0.04 and 0.68 respectively. The mean and standard deviation have thus been chosen so that the upper and lower confidence limits correspond to worst case and best case scenarios respectively ('best case' corresponding to the meeting of government targets).

1.6 Pre-exposure prophylaxis (PrEP)

In the period up to 2015, there has been no rollout of PrEP in South Africa. However, it is possible that PrEP may be introduced in future, in specific high risk groups, or in age groups in which the rate of HIV acquisition is particularly high. The uptake of PrEP is therefore assumed to depend on risk group membership or age group. The model assumes that individuals who start PrEP discontinue PrEP at a rate of 0.5 per annum [118-120].

1.6.1 Effectiveness of PrEP

Randomized controlled trials published to date have yielded conflicting estimates of the effectiveness of PrEP. We assign a beta prior distribution to represent the uncertainty regarding future average levels of PrEP effectiveness. As in our previous work [121], we set the mean and standard deviation of this distribution at 40% and 24% respectively, so that there is a wide range of possible effectiveness parameters simulated (2.5 and 97.5 percentiles are at 3% and 88% respectively). The mean corresponds to the average efficacy level in the studies of PrEP in heterosexual adults that have been published to date [122-126]. The upper limit of the confidence interval corresponds to the most optimistic estimates of PrEP efficacy in heterosexual adults; in the Partners PrEP trial, detectable levels of study drug in blood plasma were associated with efficacy levels of 86% and 90% in individuals receiving tenofovir and truvada respectively [122]. The 3% lower limit corresponds to the levels of efficacy in the FEM-PrEP trial (6% efficacy [125]) and the VOICE trial (zero efficacy [124]). The variation in efficacy is a reflection of variation in adherence, and it is possible that individuals may be more motivated to use the drugs consistently in future once their efficacy is established. In addition, new PrEP delivery methods are currently being investigated, including long-acting injectable PrEP and vaginal rings [127], which would require less frequent action on the part of the user. If proven effective and acceptable, these would lead to higher levels of adherence and hence (potentially) greater efficacy.

1.6.2 Proportionate reduction in condom usage in PrEP users

Although data from randomized trials generally do not show evidence of risk compensation in PrEP recipients [122, 123, 125], it is difficult to extrapolate from the data collected in these randomized trials, as trial participants would have been counselled on the uncertainty regarding the efficacy of the products that were being evaluated, and even if they believed the study products to be effective, would not have known whether they were receiving the study drug or the placebo. In a recent analysis of changes in behaviour after the unblinding of the Partners PrEP trial data, a statistically significant 10% increase was noted in unprotected extramarital sex, amongst individuals who were receiving open-label PrEP [119]. Similarly, in the PROUD trial, a statistically significant increase in unprotected receptive anal intercourse was observed in men who have sex with men (MSM) who were randomly allocated to receive PrEP [128]. Another recent microbicide acceptability study found that women were resistant to the idea of using both condoms and microbicides simultaneously [127]. This suggests that some reduction in condom use could occur. However, in a study of MSM and transgender women who were offered PrEP following news of its efficacy, unprotected anal intercourse declined similarly over the course of the study in those who chose to receive PrEP and those who did not take PrEP [120]. We assign a beta prior distribution to represent the uncertainty around the average percentage reduction in condom usage that occurs in users of PrEP. This distribution has a mean of 10% and a standard deviation of 10% (with 2.5 and 97.5 percentiles at 0% and 37% respectively). The mean thus corresponds to the data from the Partners PrEP trial, while the standard deviation has been chosen so that the lower limit corresponds to negligible risk compensation.

1.6.3 Time to the introduction of PrEP for sex workers

It is uncertain if and when PrEP would be promoted among sex workers. Although PrEP is not formally endorsed in the 2012-16 National Strategic Plan and in government target setting, the SANAC strategic plan for sex workers does mention the need to pilot PrEP programmes in sex workers [129], and the Department of Health has commissioned work to explore the cost-effectiveness of a PrEP promotion strategy for sex workers. The South African Medicines Control Council has not yet licensed tenofovir for use as a prophylactic agent. As with other new interventions, we assume that the time to PrEP introduction among sex workers (in years from 2015) is Weibull distributed with a median of 10 years and a shape parameter of 0.55 (implying a 30% chance that PrEP is made available to sex workers before 2018).

1.6.4 Time to the introduction of PrEP for youth aged 15-24

The timing of the introduction of PrEP for youth is modelled in the same way as that for sex workers, i.e. using a Weibull distribution with a median of 10 years to represent the uncertainty regarding the time to the promotion of PrEP for youth (after 2015).

1.6.5 Annual rate at which sex workers adopt PrEP if it is available

Few studies have investigated the acceptability of PrEP among sex workers. In a study of sex workers in four countries (Kenya, India, Peru and Ukraine), Eisingerich *et al* [130] found that more than 90% of sex workers reported that they would probably or definitely use PrEP if it was available. In another Kenyan study, 80% of sex workers and MSM reported that they would use PrEP if it was found to be effective [131]. However, stated acceptability may differ from actual uptake. Among MSM attending STI clinics in San Francisco, who were offered PrEP, only 49% accepted the offer [118]. Sex workers may avoid PrEP if they are concerned that it provides no protection against other STIs and pregnancy. Even if PrEP is highly acceptable, actual levels of uptake may be low if PrEP promotion programmes struggle to reach women engaging in commercial sex; this is likely given that commercial sex is currently criminalized in South Africa. We assign a gamma distribution to represent the uncertainty regarding the annual rate at which sex workers adopt PrEP if it is available to them. This gamma distribution has a mean of 0.3 and a standard deviation of 0.2; with this mean uptake of 0.3 per annum, the average PrEP coverage in sex workers would be approximately 26% (0.3/(0.3 + 0.5 + 1/3), given an assumed PrEP discontinuation rate of 0.5 per annum and an assumed average duration of commercial sex of 3 years). The 2.5 and 97.5 percentiles of the prior distribution (0.04 and 0.80 respectively) correspond to PrEP coverage levels of 5% and 49% respectively. The mean and standard deviation have thus been chosen in such a way that the upper bound on the confidence interval corresponds to an optimistic scenario in which all sex workers have access to PrEP and have rates of PrEP coverage similar to those observed in MSM in San Francisco who had been offered PrEP, while the mean of the distribution yields a PrEP coverage level that is roughly half of that [118].

1.6.6 Annual rate at which sexually active youth adopt PrEP if it is available

Although self-reported willingness to use PrEP among South African youth appears similar to the high levels reported among sex workers [130], actual uptake may again be very different. Although youth are an easier population to access than sex workers, there are important concerns that still need to be resolved regarding the safety of tenofovir and truvada in adolescents, especially in relation to bone mineral density. There are potentially also legal obstacles to offering PrEP to adolescents below the age of majority. We model the uncertainty regarding the likely uptake of PrEP in sexually active youth (ages 15-24) using the same approach as for sex workers, i.e. assigning a gamma prior with a mean of 0.3 to represent the annual rate at which youth would initiate PrEP if it were available.

2. Statistical analysis

The model is calibrated to historic HIV prevalence data using a two-step Bayesian procedure: the first step involves calibrating the model to adult HIV prevalence data and the second involves calibrating the model to paediatric HIV prevalence data. Once the calibration is completed, the uncertainty analysis to explore the effect of potential future determinants of HIV incidence is performed. Although prior distributions are specified for both calibration steps and for the uncertainty analysis, each step considers a different group of priors, with all other parameter values being held constant. The sections that follow describe the different steps in more detail.

2.1 Calibration step 1: Model fitting to adult HIV prevalence data

The process of fitting the model to South African HIV prevalence data is similar to that described previously [23], i.e. it is based on defining a likelihood statistic that represents the degree of consistency between age-specific model estimates of HIV prevalence and corresponding survey estimates, and finding the model parameters that yield the highest likelihood values. However, a few changes have been made to the procedure in this analysis:

- The parameters that are varied in the model-fitting procedure are limited to the sexual behaviour and HIV transmission parameters (we do not include the parameters that determine rates of HIV survival, since we are not calibrating the model to recorded death data).
- Antenatal survey data collected prior to 1997 are not included in the model fitting procedure, as the early antenatal surveys were based on convenience samples and did not follow a standard sampling procedure.
- HIV prevalence data from surveys of sex workers are included in the likelihood definition. The method for defining the likelihood in respect of the sex worker HIV prevalence data is described in the supplementary material of a previous publication [132].

Table S2.1 summarizes the prior distributions used to represent the initial uncertainty regarding each of the parameters considered in the model fitting procedure, as well as the posterior (best-fitting) values for each parameter.

Table S2.1: Comparison of prior and posterior distributions for parameters considered in calibration to adult HIV prevalence data

Parameter	Prior distribution (mean, 95% CI)	Posterior distribution (mean, 95% CI)
Mean of age adjustment function for non-spousal sex	35.0 (25.9-45.5)	38.3 (360.0-40.5)
Std deviation of age adjustment function	13.0 (7.8, 19.5)	19.1 (17.1-21.2)
Relative rate of partner acquisition in low-risk men	0.50 (0.025-0.975)	0.43 (0.24-0.67)
Relative rate of partner acquisition in low-risk women	0.50 (0.025-0.975)	0.14 (0.11-0.18)
Reduction in unprotected sex after HIV diagnosis	0.68 (0.36-0.93)	0.46 (0.33-0.58)
Female-to-male transmission probability in short-term/ non-spousal partnerships	0.0080 (0.0032-0.0149)	0.0080 (0.0071-0.0090)
Male-to-female transmission probability in short-term/ non-spousal partnerships	0.0120 (0.0043-0.0236)	0.0190 (0.0147-0.0240)
Female-to-male transmission probability in long-term/ spousal partnerships	0.0020 (0.0008-0.0037)	0.0014 (0.0009-0.0022)
Male-to-female transmission probability in long-term/ spousal partnerships	0.002 (0.0008-0.0037)	0.0042 (0.0027-0.0058)
Initial HIV prevalence in high risk women, ages 15-49, In 1985	0.00100 (0.00005-0.00195)	0.00186 (0.00155-0.00199)

2.2 Calibration step 2: Model fitting to paediatric HIV prevalence data

The process of fitting the model to South African HIV prevalence data is similar to that described previously [104], with the following modifications:

- The likelihood function has been updated to include paediatric HIV prevalence data from the most recent national household survey [57].
- The efficacy of dual therapy (single-dose nevirapine together with short-course AZT) in preventing mother-to-child transmission has been included as one of the free parameters in the model-fitting process.

Table S2.2 summarizes the prior distributions and posterior distributions for the parameters that were allowed to vary in the model fitting procedure.

Table S2.2: Comparison of prior and posterior distributions for parameters considered in calibration to paediatric HIV prevalence data

Parameter	Prior distribution (mean, 95% CI)	Posterior distribution (mean, 95% CI)
Transmission rate at/before birth, from acutely-infected women with no ARV prophylaxis	0.350 (0.203-0.514)	0.326 (0.187-0.479)
% reduction in perinatal MTCT if mother receives single-dose nevirapine + short-course zidovudine	85.0% (74.0-93.4%)	86.3% (75.8-94.4%)
Probability of MTCT from chronically-infected mothers, per year of mixed feeding	0.140 (0.095-0.192)	0.103 (0.072-0.139)
Probability of MTCT from acutely- infected mothers, per month of mixed feeding	0.16 (0.106-0.223)	0.147 (0.099-0.202)
Ratio of postnatal transmission risk per month of EBF to postnatal transmission risk per month of mixed feeding	0.500 (0.213-0.787)	0.503 (0.216-0.780)
Children infected at/before birth		
Annual rate of progression to late disease in older children	0.400 (0.229-0.619)	0.428 (0.255-0.638)
Excess annual rate of progression to late disease in neonates	2.00 (1.54-2.52)	2.11 (1.56-2.62)
Relative rate of progression to late disease if infected after birth	0.350 (0.096-0.666)	0.362 (0.147-0.666)
Children in late disease, untreated		
Annual rate of AIDS mortality in older children	0.120 (0.069-0.186)	0.143 (0.098-0.203)
Excess annual rate of AIDS mortality in neonates	3.50 (2.85-4.22)	3.64 (2.95-4.39)

ARV = antiretroviral. EBF = exclusive breastfeeding. MTCT = mother-to-child transmission.

2.3 Uncertainty analysis: determinants of future HIV incidence trends

The prior distributions chosen to represent the uncertainty regarding future epidemiological parameters are summarized in Table 1 of the main text. In most cases the prior distributions relate to the period five years after the baseline estimate for the epidemiological parameter, and the change between the baseline parameter and the parameter sampled from the prior distribution is assumed to be phased in linearly over the five-year term. However, in the following cases the change is phased in over a period of less than five years:

- For some of the parameters, phase-in is assumed to be immediate upon the adoption of a new policy. This applies to parameters 1.4, 1.6, 2.4, 6.1, 6.2, 6.5 and 6.6 in Table 1 of the main text (for these parameters there are prior distributions to represent the uncertainty regarding the timing of the new policies).
- As discussed in section 1.4.2, the introduction of WHO option B occurred at the end of 2012. The change in the fraction of HIV-positive mothers not starting ART who receive short-term ARV prophylaxis is therefore assumed to start in the 2012/13 year and reaches its ultimate level in 2013/14 (i.e. a two-year phase in).
- The change in the median duration of exclusive breastfeeding (EBF) is phased in over three years, as the phasing out of the free provision of free formula milk in public health services is believed to have been fairly rapid, and the associated change in emphasis on EBF is likely to have been correspondingly quick.
- Similarly, the change in the fraction of mothers discontinuing EBF who stop breastfeeding completely is phased in over a three year period, for the same reason.

3. Additional results

3.1 One-way sensitivity analyses

The figures below show the effect of changing each parameter from its median value to the upper and lower bounds on the 95% confidence interval around the parameter (see Table 1 of the main text for the values of the lower and upper bounds). Figure S3.1 shows the effect on the average adult HIV incidence rate over the 2015-35 period (absolute percentage change in incidence associated with each parameter change). Results are generally consistent with Figure 3 of the main text, although there are several cases where the average incidence rate is more sensitive to downside variation than to upside variation. This is particularly true for the time delays to the introduction of new interventions, which is because the full impact of these interventions is usually not realized immediately, and hence there is a non-linear relation between the time at which the intervention is introduced and its cumulative impact. The incidence rate is more sensitive to an increase in partner age differences than to a reduction in partner age differences, possibly because the increases in partner age differences are assumed to be associated with increases in the standard deviation of the partner age distribution.

Figure S3.2 shows the effects of parameter changes on the average mother-to-child transmission rate over the 2015-35 period. Results are generally consistent with Figure 3 of the main text and are consistent with the associations seen in Figure S3.1 for the adult interventions.

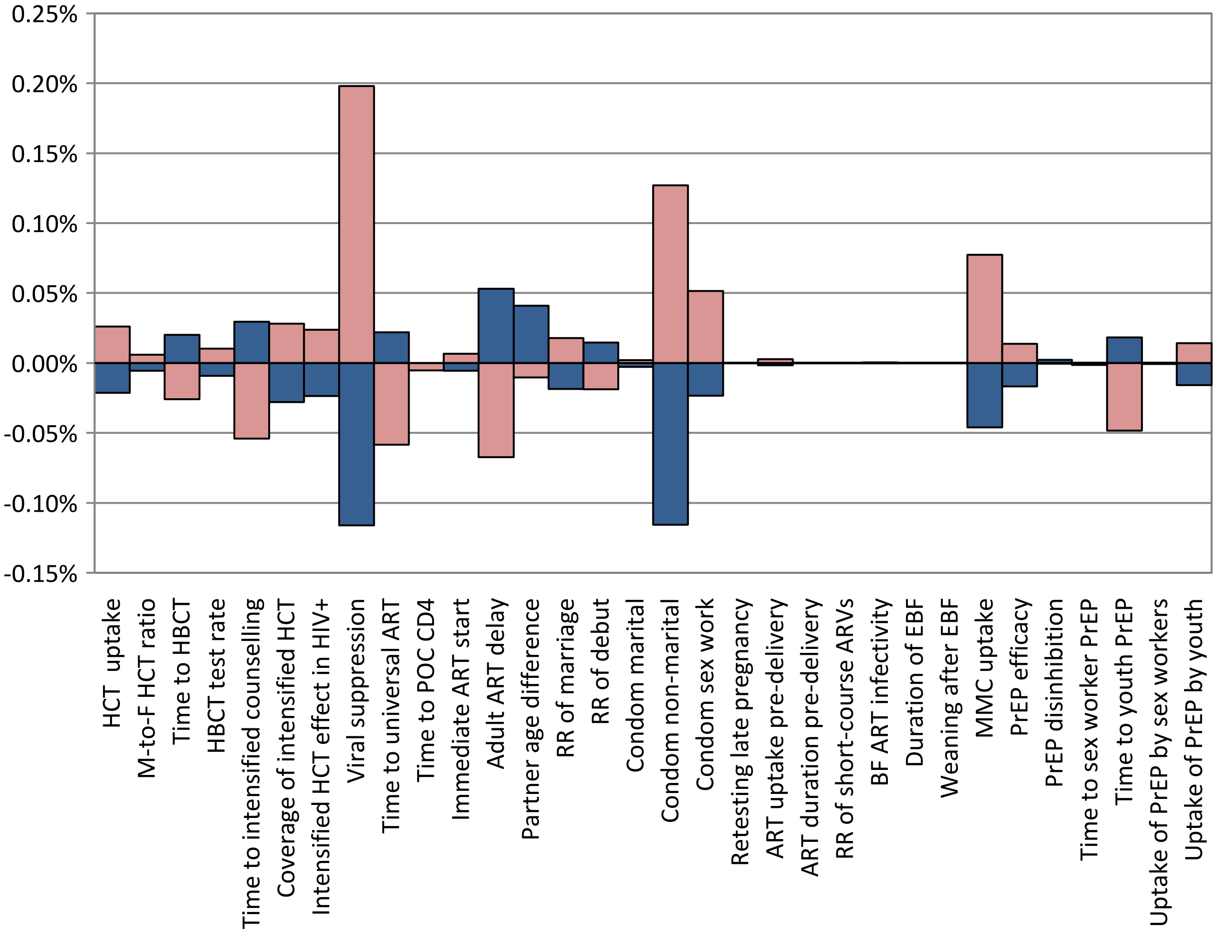

Figure S3.1: Effect on average HIV incidence in adults of changing parameter values to upper (blue) or lower (pink) limits on prior uncertainty ranges

Bars represent the absolute change in incidence, in adults aged 15-49, over the 2015-35 period. The baseline scenario corresponds to the median parameter values. ART = antiretroviral treatment, BF = breastfeeding, EBF = exclusive breastfeeding, HBCT = home-based counselling and testing, HCT = HIV counselling and testing, MMC = medical male circumcision, POC = point-of-care, PrEP = pre-exposure prophylaxis, RR = relative rate.

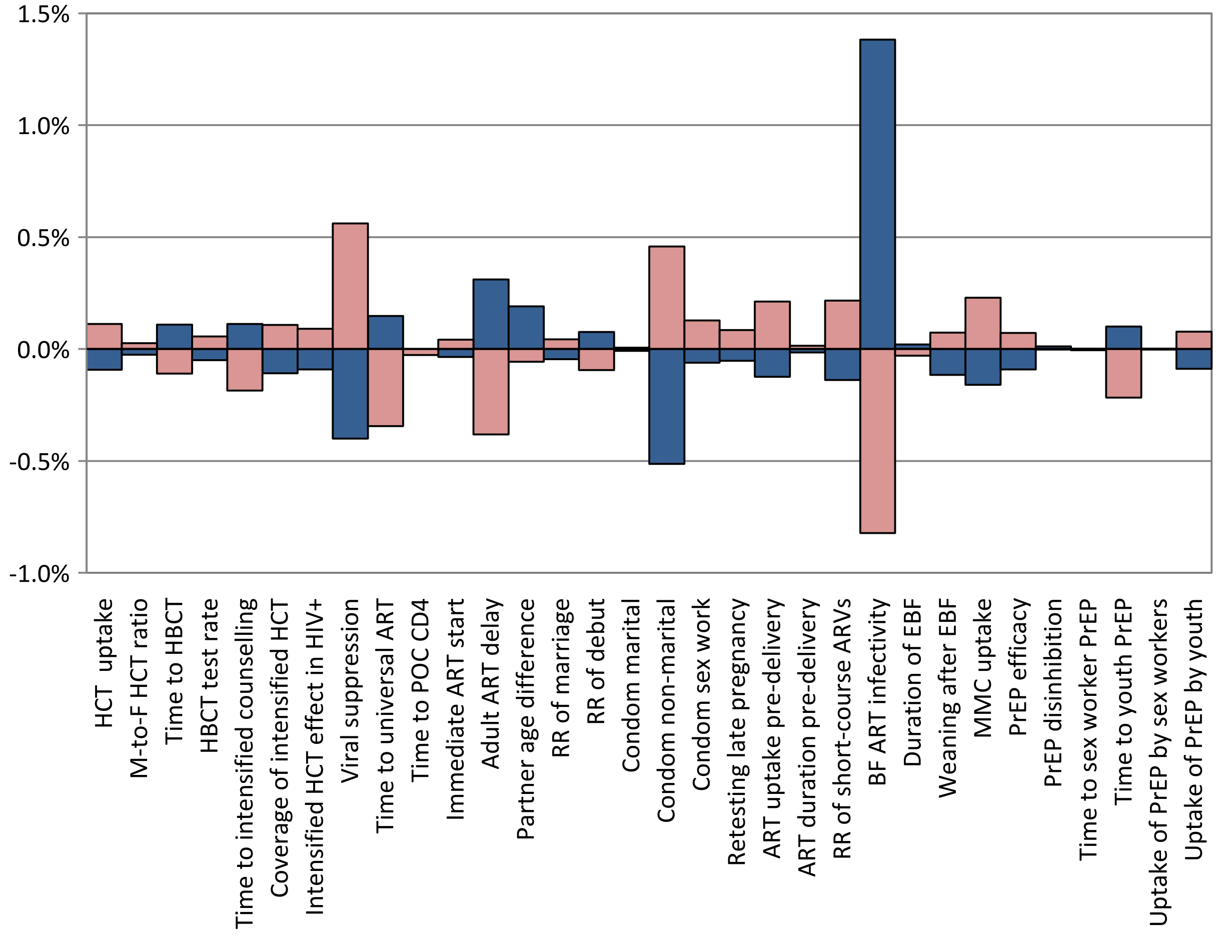

Figure S3.2: Effect on average mother-to-child transmission rates of changing parameter values to upper (blue) or lower (pink) limits on prior uncertainty ranges

Bars represent the absolute change in the mother-to-child transmission rate (including postnatal transmission and transmission from mothers who seroconvert during breastfeeding), over the 2015-35 period. The baseline scenario corresponds to the median parameter values. ART = antiretroviral treatment, BF = breastfeeding, EBF = exclusive breastfeeding, HBCT = home-based counselling and testing, HCT = HIV counselling and testing, MMC = medical male circumcision, POC = point-of-care, PrEP = pre-exposure prophylaxis, RR = relative rate.

Figure S3.3 shows the sensitivity of the fraction of HIV-positive adults who are diagnosed to the different parameters. The fraction diagnosed is sensitive to the first four parameters, since these parameters all determine the rate of HCT uptake. However, the estimates are also sensitive to many of the other parameters that influence the adult HIV incidence rate (e.g. the viral suppression rate and the rate of condom use in non-marital relationships), since a low HIV incidence rate implies a low rate of recruitment into the undiagnosed pool.

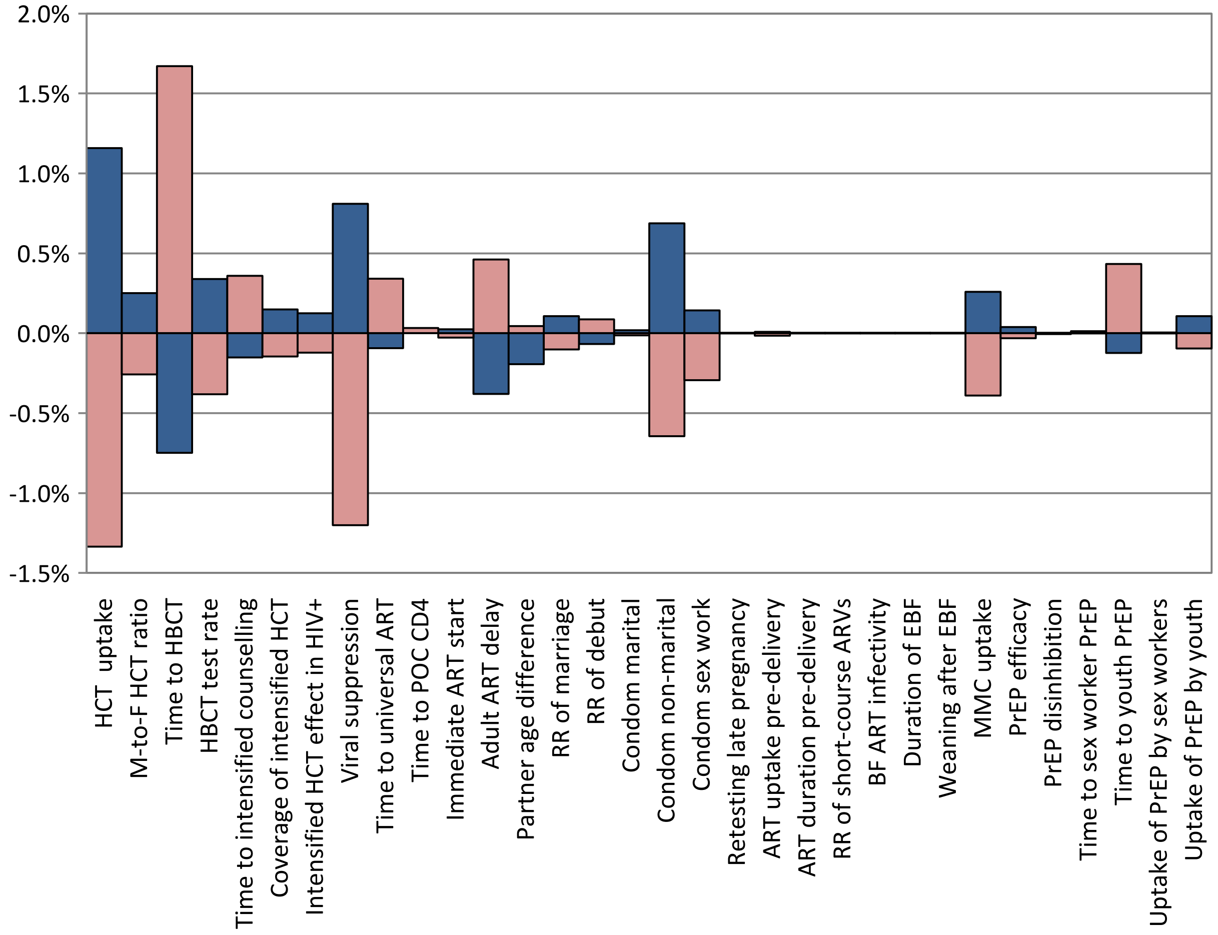

Figure S3.3: Effect on average fraction of HIV-positive adults diagnosed of changing parameter values to upper (blue) or lower (pink) limits on prior uncertainty ranges

Bars represent the absolute change in the fraction of HIV-positive adults (aged 15 and older), averaged over the 2015-35 period. The baseline scenario corresponds to the median parameter values. ART = antiretroviral treatment, BF = breastfeeding, EBF = exclusive breastfeeding, HBCT = home-based counselling and testing, HCT = HIV counselling and testing, MMC = medical male circumcision, POC = point-of-care, PrEP = pre-exposure prophylaxis, RR = relative rate.

Figure S3.4 shows the change in the fraction of HIV-diagnosed adults who are on ART as a result of changes to each of the parameter values. As might be expected, accelerating the time to universal ART eligibility and reducing average delays to ART initiation following diagnosis would have the greatest impact on the fraction of diagnosed adults on ART. However, increases in rates of HIV testing have the effect of *reducing* the level of ART coverage in the HIV-diagnosed population, because in the short term an increase in the rate of HIV testing means an increase in the number of diagnosed but untreated individuals relative to the number of treated individuals. Other parameters that influence HIV incidence (e.g. the viral suppression rate and the rate of condom use in non-marital relationships) also influence the fraction of diagnosed adults on ART because a low HIV incidence rate implies a relatively low fraction of diagnosed individuals who are recently diagnosed, and hence a relatively high fraction of diagnosed individuals who are on ART.

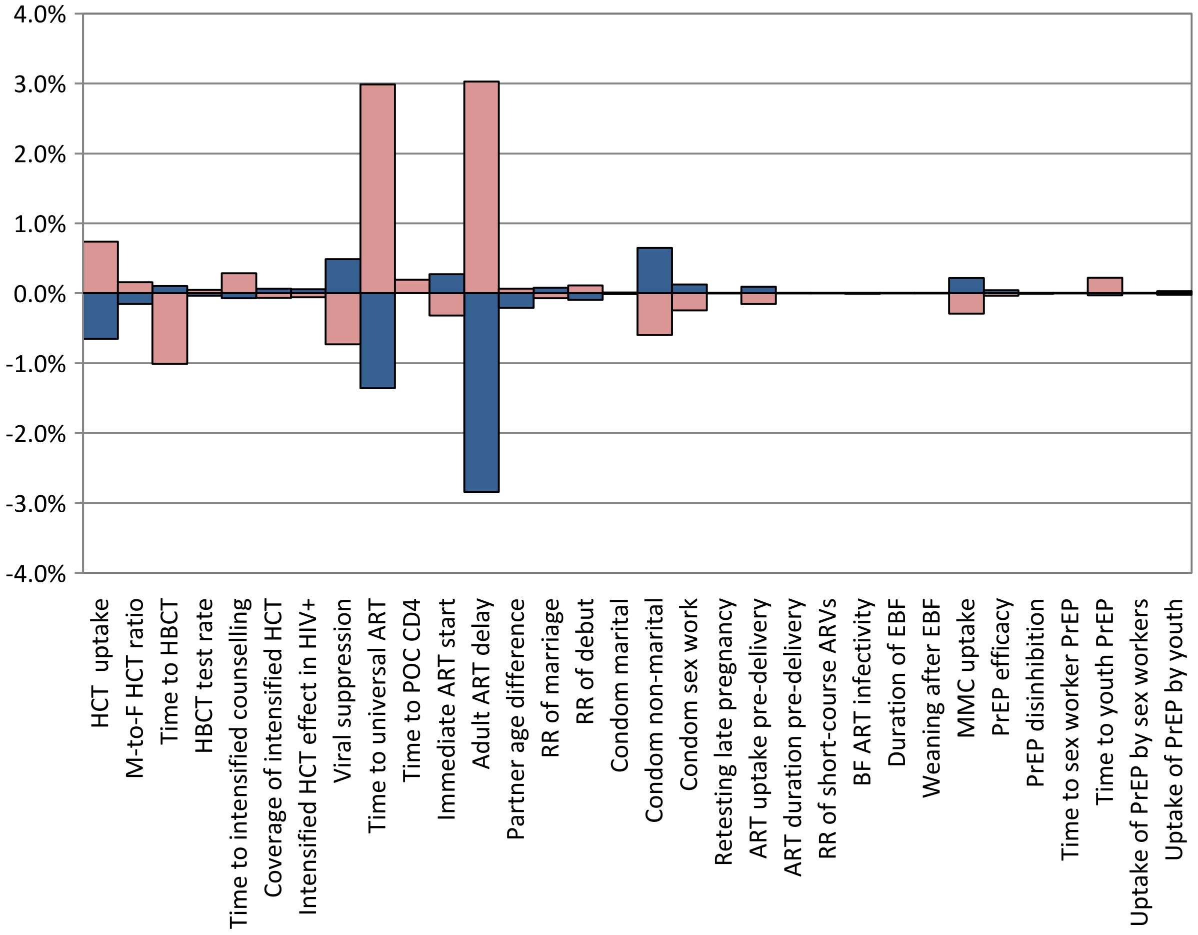

Figure S3.4: Effect on average fraction of HIV-diagnosed adults who are on ART of changing parameter values to upper (blue) or lower (pink) limits on prior uncertainty ranges

Bars represent the absolute change in the fraction of HIV-diagnosed adults (aged 15 and older) who are on ART, averaged over the 2015-35 period. The baseline scenario corresponds to the median parameter values. ART = antiretroviral treatment, BF = breastfeeding, EBF = exclusive breastfeeding, HBCT = home-based counselling and testing, HCT = HIV counselling and testing, MMC = medical male circumcision, POC = point-of-care, PrEP = pre-exposure prophylaxis, RR = relative rate.

3.2 Inclusion of uncertainty in baseline conditions

Although the results in the main text are those obtained when the model parameters shown in Tables S2.1 and S2.2 are fixed at the posterior means (i.e. not allowing for uncertainty in the baseline conditions), the analysis was repeated by randomly pairing the posterior samples summarized in Tables S2.1 and S2.2 with the parameter values sampled from the prior distributions in Table 1 of the main text. The resulting projections of key model outputs are compared with those obtained in the main analysis (Figure 2 of the main text) in Table S3.1. The confidence intervals around the mother-to-child transmission rates are slightly wider when the uncertainty regarding the baseline conditions is included, but for the adult model outputs the inclusion of the uncertainty regarding baseline conditions makes negligible difference to the confidence intervals.

Table S3.1: Comparison of model outputs with and without allowance for uncertainty in baseline conditions

	Model output in 2020† (mean, 95% CI)		Model output in 2035† (mean, 95% CI)	
	Baseline uncertainty	No baseline uncertainty	Baseline uncertainty	No baseline uncertainty
HIV incidence (15-49)	0.63% (0.36-0.97%)	0.64% (0.35-0.98%)	0.29% (0.10-0.63%)	0.29% (0.10-0.62%)
Mother-to-child transmission rate*	5.3% (3.6-7.4%)	5.2% (3.7-6.9%)	5.1% (3.3-7.3%)	4.9% (3.3-6.8%)
% of HIV-positive adults diagnosed	93.4% (88.9-97.3%)	93.4% (89.0-97.2%)	97.3% (93.8-99.0%)	97.2% (93.7-99.0%)
% of HIV-diagnosed adults on ART	82.9% (76.3-90.4%)	82.8% (76.6-90.3%)	91.1% (86.0-93.4%)	91.1% (86.1-93.4%)
% virally suppressed	79.6% (57.2-94.9%)	79.6% (57.3-94.9%)	80.4% (59.3-95.1%)	80.4% (59.3-95.1%)

* Denominator is the number of births to HIV-positive mothers plus the number of mothers who seroconvert while breastfeeding, and numerator includes all cases of postnatal transmission. † For cross-sectional measures, the output is defined at the middle of the year. For longitudinal measures (the first two measures), the output is defined at the start of the year (i.e. the average over the period from the mid-point of the preceding year to the mid-point of the stated year).

Correlation coefficients were calculated between the adult HIV parameters in Table S2.1 and the average adult HIV incidence rate over the 2015-35 period. The only parameter that was significantly associated with the incidence rate was the reduction in unprotected sex after HIV diagnosis (r=-0.18). A similar analysis was conducted to assess the association between the paediatric HIV parameters in Table S2.2 and the average mother-to-child transmission rate over the 2015-35 period. A number of the transmission parameters were strongly positively associated with the mother-to-child transmission rate, including the annual transmission probability from breastfeeding mothers in the chronic phase of HIV infection (r=0.31), the monthly breastfeeding transmission probability during the acute phase of maternal HIV infection (r=0.23) and the perinatal transmission probability if the mother seroconverts during late pregnancy (r=0.09). A number of the HIV disease progression and mortality parameters were also positively associated with the average mother-to-child transmission probability (results not shown). This is because when the model is fitted to observed HIV prevalence levels, incidence rates and mortality rates are positively correlated (i.e. it is not possible to increase the paediatric HIV incidence rate while maintaining a specified prevalence level unless the assumed mortality rates are also increased).

3.3 Inclusion of correlation between epidemiological parameters

The results presented in the main text have been calculated by sampling independently from different prior distributions. However, in reality there is likely to be a degree of dependence between certain parameters. In the sensitivity analysis presented here, we consider the effect of allowing for correlation between the parameters sampled from the different prior distributions. The following associations are assumed:

- The rate of viral suppression in adults on ART is assumed to be negatively correlated with the infectivity of breastfeeding mothers who are on ART at the time they initiate breastfeeding (r=-0.5).
- The odds of condom use in short-term relationships is assumed to be positively correlated with the odds of condom use in long-term condom use and the odds of

condom use in sex worker-client relationships (r=0.5 for all associations), since condom promotion and distribution programmes are likely to influence condom usage rates similarly across relationship types.

- The rate of first-time HIV testing in HIV-negative women is assumed to be positively correlated with the time to the introduction of home-based HCT (HBCT) (r=0.5), since it is more likely that HBCT would be introduced if there were concern about low rates of HIV testing. (The rate of first-time testing referred to here excludes testing through HBCT programmes.)
- Risk compensation in individuals using PrEP is assumed to be positively associated with efficacy of PrEP (r=0.5), since individuals who know they are receiving partially effective prevention methods may be less inclined to behavioural disinhibition than individuals who regard their prevention methods as fully protective [133].

Rank correlation is induced using the method proposed by Iman and Conover [134].

Table S3.2 shows how the confidence intervals for selected model outputs change when the above correlation levels are assumed. In general, the inclusion of the correlation makes little difference to the confidence interval widths, which is probably because the uncertainty around most outputs is driven mainly by one or two key parameters, and assumed correlations with other parameters are only of secondary importance. In the case of the fraction of HIV-positive adults who are diagnosed, the assumed correlations lead to a slight reduction in the confidence interval widths, which is probably due to the assumed negative association between HBCT and HCT uptake through other means. In the case of the mother-to-child transmission rate, the assumed correlations lead to a slight increase in the confidence interval width, due to the assumed positive association between the effect of ART on heterosexual transmission probabilities and the effect of ART on mother-to-child transmission probabilities.

Table S3.2: Comparison of model outputs with and without allowance for correlation between parameters

	Model output in 2020† (mean, 95% CI)		Model output in 2035† (mean, 95% CI)	
	Correlation included	No correlation	Correlation included	No correlation
HIV incidence (15-49)	0.63% (0.34-0.96%)	0.64% (0.35-0.98%)	0.28% (0.09-0.60%)	0.29% (0.10-0.62%)
Mother-to-child transmission rate*	5.2% (3.6-7.0%)	5.2% (3.7-6.9%)	4.9% (3.1-6.8%)	4.9% (3.3-6.8%)
% of HIV-positive adults diagnosed	93.5% (89.6-97.3%)	93.4% (89.0-97.2%)	97.3% (94.5-99.0%)	97.2% (93.7-99.0%)
% of HIV-diagnosed adults on ART	82.8% (76.1-90.1%)	82.8% (76.6-90.3%)	91.1% (86.1-93.4%)	91.1% (86.1-93.4%)
% virally suppressed	79.6% (57.3-94.9%)	79.6% (57.3-94.9%)	80.4% (59.1-95.1%)	80.4% (59.3-95.1%)

* Denominator is the number of births to HIV-positive mothers plus the number of mothers who seroconvert while breastfeeding, and numerator includes all cases of postnatal transmission. † For cross-sectional measures, the output is defined at the middle of the year. For longitudinal measures (the first two measures), the output is defined at the start of the year (i.e. the average over the period from the mid-point of the preceding year to the mid-point of the stated year).

3.4 Characteristics of scenarios in which targets are met

Table S3.3 summarizes the average parameter values in those scenarios in which the 90-90-90 and virtual elimination targets are met. Tests for statistically significant differences between parameter values in scenarios in which the targets are met and the overall mean were performed based on the Central Limit Theorem.

The first of the 90-90-90 targets is more likely to be achieved when there is a high rate of viral suppression and when the rate of HCT uptake is high. In those scenarios in which the second 90% target is met, the parameters that differ most significantly from the overall averages are the time to universal ART eligibility (average of 1.0 year after 2015) and the average time to ART initiation in adults who are previously-diagnosed but ART-naïve (average of 6.8 months at CD4 <200 cells/μl). The parameter that differs most significantly from the overall average when the third 90% target is met is the fraction of patients starting ART at CD4 <200 cells/μl who are virologically suppressed (average of 92%). Due to the small number of scenarios in which all three 90-90-90 targets are met (n=4), few parameters are significantly different in these scenarios from the averages for all scenarios, but the most significantly different parameter is again the average time to ART initiation in adults who are previously-diagnosed but ART-naïve.

The three parameters that are most significantly different in the virtual elimination of adult HIV transmission scenarios from the overall averages (p <0.005) are similar to the parameters that are most strongly correlated with HIV incidence in the main text. In the scenarios in which virtual elimination is achieved, the average rate of viral suppression (for ART patients with baseline CD4 <200) is 90.3%, the average coverage of intensified risk reduction counselling for HIV-diagnosed adults is 70.3% and the average odds of condom use in non-marital relationships (relative to that in 1998) is 5.16.

The parameters that differ most significantly (p <0.005) in the scenarios in which virtual elimination of mother-to-child transmission is achieved include the rate of viral suppression on ART (average value of 79.4%), the time to ART initiation in adults who are previously-diagnosed but ART-naïve (average value of 17.0 months), the average odds of condom use in non-marital relationships (average value of 3.32 relative to 1998), the relative risk of breastmilk transmission for mothers on ART compared to mothers not on ART (average of 0.152) and the annual uptake of MMC (average value of 0.325).

Table S3.3: Average parameter values in different scenario subsets

	All scenarios	Scenarios in which 90-90-90 targets are met				Scenarios in which virtual elimination targets are met	
		1st 90	2nd 90	3rd 90	All	Adults	MTCT
	(n=1000)	(n=929)	(n=32)	(n=139)	(n=4)	(n=21)	(n=551)
**1. HIV counselling and testing (HCT)**							
1.1 Annual rate of first-time HIV testing in women aged 25	0.250	0.255*	0.241	0.249	0.253	0.251	0.254
1.2 Ratio of male HCT uptake to female HCT uptake at age 25	0.680	0.681	0.664	0.679	0.664	0.649*	0.680
1.3 Time to the introduction of home-based HCT	33.1	31.9	24.9	34.6	22.3	46.9	28.7
1.4 Annual rate of HIV testing through home-based HCT	0.350	0.350	0.364	0.363	0.421	0.414*	0.347
1.5 Time to introduction of intensified counselling for HIV+	33.1	33.2	24.5	35.1	45.8	10.4	29.1
1.6 Coverage of intensified risk reduction counselling	0.500	0.506	0.506	0.505	0.572	0.703**	0.519
1.7 Effect of intensified counselling on unprotected sex	0.500	0.503	0.526	0.525	0.674	0.590	0.525*
**2. Adult antiretroviral treatment (ART)**							
2.1 Proportion of adult ART patients who are virally suppressed	0.770	0.779*	0.781	0.920**	0.923*	0.903**	0.794**
2.2 Time to the introduction of universal ART eligibility	33.1	32.6	1.0**	33.4	1.5	20.9	27.4*
2.3 Time to the introduction of POC CD4 testing	33.1	33.4	26.2	30.9	24.3	17.3	33.0
2.4 Fraction starting ART immediately after diagnosis	0.600	0.600	0.614	0.601	0.604	0.646*	0.604
2.5 Time to ART initiation if ART is not started on diagnosis	18.0	17.8	6.83**	17.8	6.65**	14.1*	17.0**
**3. Behaviour change**							
3.1 Average partner age difference in non-marital relationships	3.00	2.99	3.09	2.90	2.69	2.70	3.01
3.2 Ratio of marriage rates in 2016/17 to those in 2011/12	1.00	1.00	1.08*	0.98	0.99	1.07	1.00
3.3 Ratio of sexual debut rates in 2016/17 to those in 2011/12	1.00	1.00	1.00	0.99	1.09	0.98	0.99
3.4 Odds ratio: condom use in marital relationships	1.78	1.78	1.78	1.78	1.84	1.75	1.76
3.5 Odds ratio: condom use in non-marital relationships	3.14	3.19	3.38	3.18	3.63	5.16**	3.32**
3.6 Odds ratio: condom use in SW-client relationships	3.80	3.81	4.18	3.77	4.55	4.10	3.84
**4. Prevention of mother-to-child transmission (PMTCT)**							
4.1 Rate of retesting in late pregnancy	0.750	0.748	0.736	0.752	0.801	0.753	0.753
4.2 % of newly-diagnosed pregnant women who start ART	0.900	0.901	0.912	0.900	0.914	0.914	0.902
4.3 % increase in mean duration of ART prior to delivery	0.700	0.701	0.704	0.686	0.703	0.664	0.706
4.4 Relative rate of short-course ARV uptake	0.500	0.499	0.500	0.517	0.758	0.511	0.533*
4.5 Relative infectivity of breastfeeding women on ART	0.200	0.200	0.204	0.188	0.151	0.189	0.152**
4.6 Median duration of exclusive breastfeeding (EBF)	4.00	4.01	4.04	4.02	3.59	4.14	4.01
4.7 Fraction of mothers discontinuing EBF who stop BF	0.150	0.150	0.142	0.149	0.120	0.150	0.155
**5. Medical male circumcision (MMC)**							
5.1 Annual probability of MMC uptake in men	0.300	0.304	0.274	0.297	0.335	0.377*	0.325**
**6. Pre-exposure prophylaxis (PrEP)**							

6.1 Effectiveness of PrEP	0.401	0.403	0.335	0.390	0.369	0.417	0.408
6.2 Proportionate reduction in condom usage in PrEP users	0.100	0.100	0.088	0.084	0.036	0.089	0.100
6.3 Time to the introduction of PrEP for sex workers	33.1	32.9	32.2	26.7	2.3	45.4	32.1
6.4 Time to the introduction of PrEP for youth aged 15-24	33.1	33.2	33.8	38.7	11.0	21.3	31.6
6.5 Annual rate at which sex workers adopt PrEP	0.300	0.303	0.320	0.317	0.193	0.205*	0.306
6.6 Annual rate at which sexually active youth adopt PrEP	0.300	0.299	0.285	0.296	0.257	0.360	0.320*

* Significantly different from average for all scenarios, p<0.05. ** Significantly different from average for all scenarios, p<0.005.
BF = breastfeeding, MTCT = mother-to-child transmission, POC = point of care, SW = sex worker.

3.5 Differences by age and sex

Table S3.4 shows the correlation coefficients calculated for men and women in each of three age groups. Correlation coefficients are mostly similar across age and sex categories. The effect of viral suppression after ART initiation on HIV incidence is more pronounced at the older ages than at the younger ages, as the proportion of HIV-positive individuals who are treated is lower at young ages than at older ages. However, the effect of the annual rate of first-time testing is greater at younger ages because of the proportionally lower fraction of HIV-positive adults who are diagnosed at younger ages. Effects of non-marital condom usage tend to be more substantial at younger ages, since relatively low proportions of older adults are in non-marital relationships. As might be expected, changes in sexual debut and introduction of PrEP for youth most significantly affect HIV incidence in 15-24 year olds. Although a reduction in average partner age differences would significantly reduce HIV incidence in young women as well as older men, incidence rates in men aged 15-24 would significantly *increase*. MMC uptake is significantly associated with reduced HIV incidence in men and women of all ages (except women aged 50 and older), although the expected reductions are greatest in young men.

Table S3.4: Correlation coefficients between intervention parameters and average HIV incidence rates over the 2015-35 period, by age and sex

	15-24		25-49		50+	
	F	M	F	M	F	M
**1. HIV counselling and testing (HCT)**						
1.1 Annual rate of first-time HIV testing in women aged 25	-0.13	-0.13	-0.09	-0.09	-0.05	-0.05
1.2 Ratio of male HCT uptake to female HCT uptake at age 25	-0.02	0.00	-0.04	-0.02	-0.04	-0.03
1.3 Time to the introduction of home-based HCT	0.04	0.02	0.05	0.02	0.03	0.01
1.4 Annual rate of HIV testing through home-based HCT	-0.03	-0.01	-0.07	-0.06	-0.08	-0.08
1.5 Time to introduction of intensified counselling for HIV+	0.13	0.12	0.15	0.13	0.14	0.13
1.6 Coverage of intensified risk reduction counselling	-0.12	-0.09	-0.20	-0.17	-0.20	-0.19
1.7 Effect of intensified counselling on unprotected sex	-0.12	-0.11	-0.17	-0.15	-0.15	-0.14
**2. Adult antiretroviral treatment (ART)**						
2.1 % of adult ART patients who are virally suppressed	-0.39	-0.30	-0.73	-0.67	-0.90	-0.88
2.2 Time to the introduction of universal ART eligibility	0.08	0.07	0.11	0.10	0.02	0.02
2.3 Time to the introduction of POC CD4 testing	-0.05	-0.04	-0.03	-0.04	-0.01	-0.02
2.4 Fraction starting ART immediately after diagnosis	-0.07	-0.04	-0.09	-0.07	-0.06	-0.06
2.5 Time to ART initiation if ART is not started on diagnosis	0.14	0.11	0.28	0.25	0.05	0.11
**3. Behaviour change**						
3.1 Average partner age difference in non-marital relationships	0.18	-0.12	0.09	0.12	0.00	0.19
3.2 Ratio of marriage rates in 2016/17 to those in 2011/12	0.00	0.02	-0.09	-0.12	0.00	-0.06
3.3 Ratio of sexual debut rates in 2016/17 to those in 2011/12	0.16	0.18	0.03	0.06	0.02	0.03
3.4 Odds ratio: condom use in marital relationships	0.01	0.01	-0.02	0.00	-0.02	-0.01
3.5 Odds ratio: condom use in non-marital relationships	-0.52	-0.55	-0.19	-0.25	-0.06	-0.06
3.6 Odds ratio: condom use in SW-client relationships	-0.05	-0.04	-0.05	-0.17	0.03	0.01
**4. Prevention of mother-to-child transmission (PMTCT)**						
4.1 Rate of retesting in late pregnancy	0.03	0.04	0.01	0.02	0.01	0.01
4.2 % of newly-diagnosed pregnant women who start ART	-0.06	-0.06	-0.06	-0.08	-0.05	-0.05
4.3 % increase in mean duration of ART prior to delivery	0.00	0.02	-0.01	-0.01	-0.03	-0.03
4.4 Relative rate of short-course ARV uptake	-0.01	-0.01	0.01	-0.01	0.01	0.00
4.5 Relative infectivity of breastfeeding women on ART	0.02	0.03	0.01	0.00	0.00	0.00
4.6 Median duration of exclusive breastfeeding (EBF)	-0.01	-0.01	-0.03	-0.02	-0.02	-0.02
4.7 Fraction of mothers discontinuing EBF who stop BF	-0.02	-0.04	-0.04	-0.03	-0.04	-0.03
**5. Medical male circumcision (MMC)**						
5.1 Annual probability of MMC uptake in men	-0.26	-0.37	-0.08	-0.25	-0.01	-0.12
**6. Pre-exposure prophylaxis (PrEP)**						
6.1 Effectiveness of PrEP	-0.08	-0.07	0.01	0.00	-0.01	0.00
6.2 Proportionate reduction in condom usage in PrEP users	0.10	0.11	0.05	0.07	0.04	0.04
6.3 Time to the introduction of PrEP for sex workers	0.06	0.04	0.07	0.07	0.05	0.06
6.4 Time to the introduction of PrEP for youth aged 15-24	0.11	0.10	-0.01	0.02	-0.05	-0.04
6.5 Annual rate at which sex workers adopt PrEP	-0.03	-0.04	-0.03	-0.05	-0.03	-0.04
6.6 Annual rate at which sexually active youth adopt PrEP	-0.08	-0.08	0.02	0.00	0.04	0.04

Correlation coefficients greater than 0.06 or less than -0.06 can be considered statistically significant at the 5% significance level. BF = breastfeeding, POC = point of care, SW = sex worker.

References

1. Johnson LF, Rehle TM, Jooste S, Bekker LG. Rates of HIV testing and diagnosis in South Africa, 2002-2012: successes and challenges. *AIDS* 2015; **29**:1401-1409.
2. Department of Health. Annual Performance Plan: 2014/15 - 2016/17. 2014. Available: http://www.health.gov.za/docs/strategic/2013/app201415.pdf. Accessed 20 Aug 2014
3. Department of Health. Annual Report 2013-2014. 2014. Available: http://www.health.gov.za/annualreports.php. Accessed 25 Jan 2015
4. Do M, Kincaid DL, Figueroa ME. Impacts of four communication programs on HIV testing behavior in South Africa. *AIDS Care* 2014; **26**:1109-1117.
5. Pant Pai N, Sharma J, Shivkumar S, Pillay S, Vadnais C, Joseph L*, et al.* Supervised and unsupervised self-testing for HIV in high- and low-risk populations: a systematic review. *PLoS Medicine* 2013; **10**:e1001414.
6. Maughan-Brown B, Lloyd N, Bor J, Venkataramani A. Increasing access to HIV testing: Impacts on equity of coverage and uptake from a national campaign in South Africa. Cape Town: Southern African Labour and Development Research Unit, University of Cape Town; 2015. Available: http://www.opensaldru.uct.ac.za/bitstream/handle/11090/778/2015_145_Saldruwp.pdf?sequence=1. Accessed 24 Aug 2015
7. Nglazi MD, van Schaik N, Kranzer K, Lawn SD, Wood R, Bekker LG. An incentivized HIV counseling and testing program targeting hard-to-reach unemployed men in Cape Town, South Africa. *Journal of Acquired Immune Deficiency Syndromes* 2012; **59**:e28-34.
8. van Schaik N, Kranzer K, Wood R, Bekker LG. Earlier HIV diagnosis - are mobile services the answer? *South African Medical Journal* 2010; **100**:671-674.
9. Mabuto T, Latka MH, Kuwane B, Churchyard GJ, Charalambous S, Hoffmann CJ. Four models of HIV counseling and testing: utilization and test results in South Africa. *PLoS One* 2014; **9**:e102267.
10. van Rooyen H, McGrath N, Chirowodza A, Joseph P, Fiamma A, Gray G*, et al.* Mobile VCT: reaching men and young people in urban and rural South African pilot studies (NIMH Project Accept, HPTN 043). *AIDS and Behavior* 2013; **17**:2946-2953.
11. Hensen B, Taoka S, Lewis JJ, Weiss HA, Hargreaves J. Systematic review of strategies to increase men's HIV-testing in sub-Saharan Africa. *AIDS* 2014; **28**:2133-2145.
12. Siu GE, Wight D, Seeley JA. Masculinity, social context and HIV testing: an ethnographic study of men in Busia district, rural eastern Uganda. *BMC Public Health* 2014; **14**:33.
13. Sabapathy K, Van den Bergh R, Fidler S, Hayes R, Ford N. Uptake of home-based voluntary HIV testing in sub-Saharan Africa: a systematic review and meta-analysis. *PLoS Medicine* 2012; **9**:e1001351.
14. Doherty T, Tabana H, Jackson D, Naik R, Zembe W, Lombard C*, et al.* Effect of home based HIV counselling and testing intervention in rural South Africa: cluster randomised trial. *British Medical Journal* 2013; **346**:f3481.
15. Cremin I, Nyamukapa C, Sherr L, Hallett TB, Chawira G, Cauchemez S*, et al.* Patterns of self-reported behaviour change associated with receiving voluntary

counselling and testing in a longitudinal study from Manicaland, Zimbabwe. *AIDS and Behavior* 2010; **14**:708-715.
16. Mwangi M, Bunnell R, Nyoka R, Gichangi A, Makokha E, Kim A, *et al.* Unsafe sex among HIV-infected adults in Kenya: results of a nationally representative survey. *Journal of Acquired Immune Deficiency Syndromes* 2011; **58**:80-88.
17. Voluntary HIV-1 Counselling and Testing Efficacy Study Group. Efficacy of voluntary HIV-1 counselling and testing in individuals and couples in Kenya, Tanzania and Trinidad: a randomised trial. *Lancet* 2000; **356**:103-112.
18. Müller O, Sarangbin S, Ruxrungtham K, Sittitrai W, Phanuphak P. Sexual risk behaviour reduction associated with voluntary HIV counselling and testing in HIV infected patients in Thailand. *AIDS Care* 1995; **7**:567-572.
19. Cornman DH, Kiene SM, Christie S, Fisher WA, Shuper PA, Pillay S, *et al.* Clinic-based intervention reduces unprotected sexual behavior among HIV-infected patients in KwaZulu-Natal, South Africa: results of a pilot study. *Journal of Acquired Immune Deficiency Syndromes* 2008; **48**:553-560.
20. Fisher JD, Cornman DH, Shuper PA, Christie S, Pillay S, Macdonald S, *et al.* HIV prevention counseling intervention delivered during routine clinical care reduces HIV risk behavior in HIV-infected South Africans receiving antiretroviral therapy: the Izindlela Zokuphila/Options for Health randomized trial. *Journal of Acquired Immune Deficiency Syndromes* 2014; **67**:499-507.
21. Saleh-Onoya D, Reddy PS, Ruiter RAC, Sifunda S, Wingood G, Van den Borne B. Condom use promotion among isiXhosa speaking women living with HIV in the Western Cape province, South Africa: a pilot study. *AIDS Care* 2009; **21**:817-825.
22. Brown LB, Miller WC, Kamanga G, Nyirenda N, Mmodzi P, Pettifor A, *et al.* HIV partner notification is effective and feasible in sub-Saharan Africa: opportunities for HIV treatment and prevention. *Journal of Acquired Immune Deficiency Syndromes* 2011; **56**:437-442.
23. Johnson L. THEMBISA version 1.0: A model for evaluating the impact of HIV/AIDS in South Africa. Centre for Infectious Disease Epidemiology and Research, University of Cape Town; 2014. Available: http://www.publichealth.uct.ac.za/publication-reports-0. Accessed 1 Dec 2014
24. Kranzer K, Lawn SD, Johnson LF, Bekker LG, Wood R. Community viral load and CD4 count distribution among people living with HIV in a South African township: implications for treatment as prevention. *Journal of Acquired Immune Deficiency Syndromes* 2013; **63**:498-505.
25. Huerga H, Maman D, Etard JF, Farhat JB, Bouhenia M. Mbongolwane and Eshowe HIV Impact in Population Survey. Epicentre and Medecins sans Frontieres; 2014.
26. Cornell M, Grimsrud A, Fairall L, Fox MP, van Cutsem G, Giddy J, *et al.* Temporal changes in programme outcomes among adult patients initiating antiretroviral therapy across South Africa, 2002-2007. *AIDS* 2010; **24**:2263-2270.
27. Department of Health. Health Indicators Update: Antiretroviral Indicators. 2013. Available: http://www.health.gov.za/reports.php. Accessed 14 May 2014
28. Takuva S, Brown A, Macleod W, Pillay Y, Delpech V, Puren AJ. Disparities in engagement within HIV care in South Africa [Abstract 154]. *Conference on Retroviruses and Opportunistic Infections*. Seattle, USA; 2015.
29. Fraser C, Hollingsworth TD, Chapman R, de Wolf F, Hanage WP. Variation in HIV-1 set-point viral load: epidemiological analysis and an evolutionary hypothesis. *Proceedings of the National Academy of Sciences U S A* 2007; **104**:17441-17446.

30. Quinn T, Wawer M, Sewankambo N, Serwadda D, Li C, Wabwire-Mangen F, *et al.* Viral load and heterosexual transmission of human immunodeficiency virus type 1. *New England Journal of Medicine* 2000; **342**:921-929.
31. Hughes JP, Baeten JM, Lingappa JR, Magaret AS, Wald A, de Bruyn G, *et al.* Determinants of per-coital-act HIV-1 infectivity among African HIV-1-serodiscordant couples. *Journal of Infectious Diseases* 2012; **205**:358-365.
32. Fideli US, Allen SA, Musonda R, Trask S, Hahn BH, Weiss H, *et al.* Virologic and immunologic determinants of heterosexual transmission of human immunodeficiency virus type 1 in Africa. *AIDS Research and Human Retroviruses* 2001; **17**:901-910.
33. Cohen MS, Chen YQ, McCauley M, Gamble T, Hosseinipour MC, Kumarasamy N, *et al.* Prevention of HIV-1 infection with early antiretroviral therapy. *New England Journal of Medicine* 2011; **365**:493-505.
34. Donnell D, Baeten JM, Kiarie J, Thomas KK, Stevens W, Cohen CR, *et al.* Heterosexual HIV-1 transmission after initiation of antiretroviral therapy: a prospective cohort analysis. *Lancet* 2010; **375**:2092-2098.
35. Anglemyer A, Rutherford GW, Horvath T, Baggaley RC, Egger M, Siegfried N. Antiretroviral therapy for prevention of HIV transmission in HIV-discordant couples. *Cochrane Database of Systematic Reviews* 2013; **4**:CD009153.
36. Auvert B, Males S, Puren A, Taljaard D, Carael M, Williams B. Can highly active antiretroviral therapy reduce the spread of HIV? A study in a township of South Africa. *Journal of Acquired Immune Deficiency Syndromes* 2004; **36**:613-621.
37. Fox MP, Cutsem GV, Giddy J, Maskew M, Keiser O, Prozesky H, *et al.* Rates and predictors of failure of first-line antiretroviral therapy and switch to second-line ART in South Africa. *Journal of Acquired Immune Deficiency Syndromes* 2012; **60**:428-437.
38. Cornell M, Schomaker M, Garone D, Giddy J, Hoffmann CJ, Lessells R, *et al.* Gender differences in survival among adult patients starting antiretroviral therapy in South Africa: a multicentre cohort study. *PLoS Medicine* 2012; **9**:e1001304.
39. Mills EJ, Lester R, Thorlund K, Lorenzi M, Muldoon K, Kanters S, *et al.* Interventions to promote adherence to antiretroviral therapy in Africa: a network meta-analysis. *Lancet HIV* 2014; **1**:e104-111.
40. Stop Stock Outs Project. Stock outs in South Africa: a national crisis. 2013. Available: http://stockouts.org/uploads/3/3/1/1/3311088/stop_stockouts_report_2013pdf_1.pdf. Accessed 28 April 2014
41. Phillips A, Cambiano V, Miners A, Revill P, Pillay D, Lundgren JD, *et al.* Effectiveness and cost-effectiveness of potential responses to future high levels of transmitted HIV drug resistance in antiretroviral drug-naive populations beginning treatment: modelling study and economic analysis. *Lancet HIV* 2014; **1**:e85-93.
42. Cambiano V, Bertagnolio S, Jordan MR, Pillay D, Perriëns JH, Venter F, *et al.* Predicted levels of HIV drug resistance: potential impact of expanding diagnosis, retention, and eligibility criteria for antiretroviral therapy initiation. *AIDS* 2014; **28 (Suppl 1)**:S15-23.
43. INSIGHT START Study Group. Initiation of antiretroviral therapy in early asymptomatic HIV infection. *New England Journal of Medicine* 2015; **[In press]**.
44. TEMPRANO ANRS 12136 Study Group. A trial of early antiretrovirals and isoniazid preventive therapy in Africa. *New England Journal of Medicine* 2015; **[In press]**.
45. Rosen S, Fox MP. Retention in HIV care between testing and treatment in sub-Saharan Africa: a systematic review. *PLoS Medicine* 2011; **8**:e1001056.

46. Larson BA, Schnippel K, Ndibongo B, Xulu T, Brennan A, Long L, *et al.* Rapid point-of-care CD4 testing at mobile HIV testing sites to increase linkage to care: an evaluation of a pilot program in South Africa. *Journal of Acquired Immune Deficiency Syndromes* 2012; **61**:e13-17.
47. Govindasamy D, Kranzer K, van Schaik N, Noubary F, Wood R, Walensky RP, *et al.* Linkage to HIV, TB and non-communicable disease care from a mobile testing unit in Cape Town, South Africa. *PLoS One* 2013; **8**:e80017.
48. Mujugira A, Celum C, Thomas KK, Farquhar C, Mugo N, Katabira E, *et al.* Delay of antiretroviral therapy initiation is common in East African HIV-infected individuals in serodiscordant partnerships. *Journal of Acquired Immune Deficiency Syndromes* 2014; **66**:436-442.
49. Lessells RJ, Mutevedzi PC, Cooke GS, Newell ML. Retention in HIV care for individuals not yet eligible for antiretroviral therapy: rural KwaZulu-Natal, South Africa. *Journal of Acquired Immune Deficiency Syndromes* 2011; **56**:e79-86.
50. Larson BA, Brennan A, McNamara L, Long L, Rosen S, Sanne I, *et al.* Early loss to follow up after enrolment in pre-ART care at a large public clinic in Johannesburg, South Africa. *Tropical Medicine and International Health* 2010; **15 (Suppl 1)**:43-47.
51. Fourie P, Swart C. South Africa's future AIDS governance: a focused elite survey. *Development* 2014; **56**:511-517.
52. Williams B, Gilgen D, Campbell C, Taljaard D, MacPhail C. *The natural history of HIV / AIDS in South Africa: A biomedical and social survey in Carletonville*. Johannesburg: Council for Scientific and Industrial Research; 2000.
53. Hallman K. Socioeconomic Disadvantage and Unsafe Sexual Behaviors among Young Women and Men in South Africa. New York: Population Council, Policy Research Division; 2004.
54. Shisana O, Rehle T, Simbayi LC, Parker W, Zuma K, Bhana A, *et al.* South African National HIV Prevalence, HIV Incidence, Behaviours and Communication Survey, 2005. Cape Town: HSRC Press; 2005. Available: http://www.hsrcpress.ac.za. Accessed 1 Dec 2005
55. Shisana O, Rehle T, Simbayi LC, Zuma K, Jooste S, Pillay-van Wyk V, *et al.* South African national HIV prevalence, incidence, behaviour and communication survey, 2008: A turning tide among teenagers? Cape Town: Human Sciences Research Council; 2009. Available: http://www.hsrcpress.ac.za. Accessed 9 June 2009
56. Kelly K. Communicating for action: A contextual evaluation of youth responses to HIV/AIDS. Department of Health; 2000. Available: http://www.cadre.org.za. Accessed 12 October 2006
57. Shisana O, Rehle T, Simbayi LC, Zuma K, Jooste S, Zungu N, *et al.* South African National HIV Prevalence, Incidence, and Behaviour Survey, 2012. Cape Town: Human Sciences Research Council; 2014. Available: http://www.hsrc.ac.za/en/research-outputs/view/6871. Accessed 16 April 2014
58. Johnson S, Kincaid DL, Figueroa ME, Delate R, Mahlasela L, Magni S. The Third National HIV Communication Survey, 2012. Pretoria: Johns Hopkins Health and Education in South Africa; 2013. Available: http://jhhesa.org/sites/default/files/hiv_survey.pdf. Accessed 19 April 2014
59. Johnson S, Kincaid L, Laurence S, Chikwava F, Delate R, Mahlasela L. Second National HIV Communication Survey 2009. Pretoria: Johns Hopkins Health and Education in South Africa; 2010. Available: http://jhhesa.org.za/docs/NCS_2009.pdf. Accessed 8 March 2011

60. McGrath N, Eaton JW, Bärnighausen TW, Tanser F, Newell ML. Sexual behaviour in a rural high HIV prevalence South African community: time trends in the antiretroviral treatment era. *AIDS* 2013; **27**:2461-2470.
61. Cluver L, Boyes M, Orkin M, Pantelic M, Molwena T, Sherr L. Child-focused state cash transfers and adolescent risk of HIV infection in South Africa: a propensity-score-matched case-control study. *Lancet Global Health* 2013; **1**:e362-370.
62. Baird SJ, Garfein RS, McIntosh CT, Özler B. Effect of a cash transfer programme for schooling on prevalence of HIV and herpes simplex type 2 in Malawi: a cluster randomised trial. *Lancet* 2012; **379**:1320-1329.
63. Mhongo C, Budlender D. Declining rates of marriage in South Africa: what do the number and analysts say? In: *Marriage, Land and Custom: Essays on Law and Social Change in South Africa*. Edited by Claassens A, Smythe D: Juta; 2013. pp. 181-196.
64. Posel D, Rudwick S. Changing patterns of marriage and cohabitation in South Africa. In: *Marriage, Land and Custom: Essays on Law and Social Change in South Africa*. Edited by Claassens A, Smythe D: Juta; 2013. pp. 169-180.
65. Hosegood V, McGrath N, Moultrie T. Dispensing with marriage: Marital and partnership trends in rural KwaZulu-Natal, South Africa 2000-2006. *Demographic Research* 2009; **20**:279-312.
66. Garenne M. Age at marriage and modernisation in sub-Saharan Africa. *Southern African Journal of Demography* 2004; **9**:59-79.
67. Dinkelman T, Lam D, Leibbrandt M. Household and community income, economic shocks and risky sexual behavior of young adults: evidence from the Cape Area Panel Study 2002 and 2005. *AIDS* 2007; **21 (Suppl 7)**:S49-56.
68. Hargreaves JR, Bonell CP, Morison LA, Kim JC, Phetla G, Porter JD, *et al.* Explaining continued high HIV prevalence in South Africa: socioeconomic factors, HIV incidence and sexual behaviour change among a rural cohort, 2001-2004. *AIDS* 2007; **21 (Suppl 7)**:S39-48.
69. Asiimwe-Okiror G, Opio AA, Musinguzi J, Madraa E, Tembo G, Carael M. Change in sexual behaviour and decline in HIV infection among young pregnant women in urban Uganda. *AIDS* 1997; **11**:1757-1763.
70. Cho H, Hallfors DD, Mbai, II, Itindi J, Milimo BW, Halpern CT, *et al.* Keeping adolescent orphans in school to prevent human immunodeficiency virus infection: evidence from a randomized controlled trial in Kenya. *Journal of Adolescent Health* 2011; **48**:523-526.
71. Scalway T. Presenting the evidence for social and behavioural communication. Johns Hopkins Health and Education in South Africa; 2010. Available: http://www.iphc.org.uk/Presenting%20evidence%20social%20behavioral%20communication.pdf. Accessed 4 March 2011
72. Cohen CR, Montandon M, Carrico AW, Shiboski S, Bostrom A, Obure A, *et al.* Association of attitudes and beliefs towards antiretroviral therapy with HIV-seroprevalence in the general population of Kisumu, Kenya. *PLoS One* 2009; **4**:e4573.
73. de Walque D, Kazianga H, Over M. Antiretroviral therapy perceived efficacy and risky sexual behaviors: evidence from Mozambique. *Economic Development and Cultural Change* 2012; **61**:97-125.
74. Shafer LA, Nsubuga RN, White R, Mayanja BN, Chapman R, O'Brien K, *et al.* Antiretroviral therapy and sexual behavior in Uganda: a cohort study. *AIDS* 2011; **25**:671-678.

75. Kincaid DL, Parker W, Johnson S, Schierhout G, Kelly K, Connolly C, *et al.* AIDS Communication Programmes, HIV Prevention, and Living with HIV and AIDS in South Africa, 2006. Pretoria: Johns Hopkins Health and Education in South Africa; 2008. Available: http://jhhesa.org/PDF/National%20Comm%20Report.pdf. Accessed 8 March 2011
76. Magnani R, Macintyre K, Karim AM, Brown L, Hutchinson P. The impact of life skills education on adolescent sexual risk behaviors in KwaZulu-Natal, South Africa. *Journal of Adolescent Health* 2005; **36**:289-304.
77. Kerrigan D, Kennedy CE, Morgan-Thomas R, Reza-Paul S, Mwangi P, Win KT, *et al.* A community empowerment approach to the HIV response among sex workers: effectiveness, challenges, and considerations for implementation and scale-up. *Lancet* 2015; **385**:172-185.
78. Richter ML, Chersich M, Temmerman M, Luchters S. Characteristics, sexual behaviour and risk factors of female, male and transgender sex workers in South Africa. *South African Medical Journal* 2013; **103**:246-251.
79. Shannon K, Strathdee SA, Goldenberg SM, Duff P, Mwangi P, Rusakova M, *et al.* Global epidemiology of HIV among female sex workers: influence of structural determinants. *Lancet* 2014; **385**:55-71.
80. Johnson LF, Stinson K, Newell ML, Bland RM, Moultrie H, Davies MA, *et al.* The contribution of maternal HIV seroconversion during late pregnancy and breastfeeding to mother-to-child transmission of HIV. *Journal of Acquired Immune Deficiency Syndromes* 2012; **59**:417-425.
81. Massyn N, Day C, Barron P, Haynes R, English R, Padarath A. District Health Barometer 2011/12. Durban: Health Systems Trust; 2013. Available: http://www.hst.org.za/publications/district-health-barometer-201112. Accessed 23 Oct 2013
82. Orie EF, Songca PP, Moodley J. An audit of PMTCT services at a regional hospital in South Africa. *South African Family Practice* 2009; **51**:492-495.
83. Grimwood A, Fatti G, Mothibi E, Eley B, Jackson D. Progress of preventing mother-to-child transmission of HIV at primary healthcare facilities and district hospitals in three South African provinces. *South African Medical Journal* 2012; **102**:81-83.
84. Department of Health. Annual Health Statistics 2012. 2013. Available: http://www.health.gov.za/docs/reports/2013/AnnualHealthStatisticsPublicationWeb.pdf. Accessed 16 April 2014
85. Stinson K, Giddy J, Cox V, Burton R, Ibeto M, Van Cutsem G, *et al.* Reflections on a decade of delivering PMTCT in Khayelitsha, South Africa. *Southern African Journal of HIV Medicine* 2014; **15**:30-32.
86. Hoffman RM, Black V, Technau K, van der Merwe KJ, Currier J, Coovadia A, *et al.* Effects of highly active antiretroviral therapy duration and regimen on risk for mother-to-child transmission of HIV in Johannesburg, South Africa. *Journal of Acquired Immune Deficiency Syndromes* 2010; **54**:35-41.
87. Fitzgerald FC, Bekker LG, Kaplan R, Myer L, Lawn SD, Wood R. Mother-to-child transmission of HIV in a community-based antiretroviral clinic in South Africa. *South African Medical Journal* 2010; **100**:827-831.
88. Bera E, Jwacu N, Pauls F, Mancotywa T, Ngcelwane N, Hlati Y. Risk factors for perinatal HIV-1 transmission in pregnant women requiring lifelong antiretroviral therapy: A longitudinal study at a tertiary hospital in South Africa. *South African Journal of Obstetrics and Gynaecology* 2010; **16**:6-13.

89. Van Schalkwyk M, Andersson MI, Zeier MD, La Grange M, Taljaard JJ, Theron GB. The impact of revised PMTCT guidelines: a view from a public sector ARV clinic in Cape Town, South Africa. *Journal of Acquired Immune Deficiency Syndromes* 2013; **63**:234-238.
90. Homsy J, Bunnell R, Moore D, King R, Malamba S, Nakityo R, *et al.* Reproductive intentions and outcomes among women on antiretroviral therapy in rural Uganda: a prospective cohort study. *PLoS One* 2009; **4**:e4149.
91. Tonwe-Gold B, Ekouevi DK, Viho I, Amani-Bosse C, Toure S, Coffie PA, *et al.* Antiretroviral treatment and prevention of peripartum and postnatal HIV transmission in West Africa: evaluation of a two-tiered approach. *PLoS Medicine* 2007; **4**:e257.
92. Shapiro RL, Hughes MD, Ogwu A, Kitch D, Lockman S, Moffat C, *et al.* Antiretroviral regimens in pregnancy and breast-feeding in Botswana. *New England Journal of Medicine* 2010; **362**:2282-2294.
93. Peltier CA, Ndayisaba GF, Lepage P, van Griensven J, Leroy V, Pharm CO, *et al.* Breastfeeding with maternal antiretroviral therapy or formula feeding to prevent HIV postnatal mother-to-child transmission in Rwanda. *AIDS* 2009; **23**:2415-2423.
94. Palombi L, Marazzi MC, Voetberg A, Magid NA. Treatment acceleration program and the experience of the DREAM program in prevention of mother-to-child transmission of HIV. *AIDS* 2007; **21 (Suppl 4)**:S65-71.
95. Geddes R, Giddy J, Butler LM, Van Wyk E, Crankshaw T, Esterhuizen TM, *et al.* Dual and triple therapy to prevent mother-to-child transmission of HIV in a resource-limited setting - lessons from a South African programme. *South African Medical Journal* 2011; **101**:651-654.
96. Kesho Bora Study Group. Triple antiretroviral compared with zidovudine and single-dose nevirapine prophylaxis during pregnancy and breastfeeding for prevention of mother-to-child transmission of HIV-1 (Kesho Bora study): a randomised controlled trial. *Lancet Infectious Diseases* 2011; **11**:171-180.
97. Department of Health. Clinical Guidelines: PMTCT (Prevention of Mother-to-Child Transmission). 2010. Available: http://www.rhru.co.za/Resources/Documents/2010%20PMTCT%20Guidelines.pdf. Accessed 7 June 2010
98. Fatti G, Shaikh N, Eley B, Jackson D, Grimwood A. Adolescent and young pregnant women at increased risk of mother-to-child transmission of HIV and poorer maternal and infant health outcomes: a cohort study at public health facilities in the Nelson Mandela Bay Metropolitan district, Eastern Cape, South Africa. *South African Medical Journal* 2014; **104**:874-880.
99. Stinson K, Jennings K, Myer L. Integration of antiretroviral therapy services into antenatal care increases treatment initiation during pregnancy: a cohort study. *PLoS One* 2013; **8**:e63328.
100. Western Cape Department of Health. PMTCT Clinical Guidelines Update. 2013. Available: http://www.westerncape.gov.za/assets/departments/health/wcp_2013_pmtct_clinical_guidelines_update_final_replacement_2.pdf. Accessed 28 Oct 2014
101. Department of Health. National consolidated guidelines for the prevention of mother-to-child transmission of HIV and the management of HIV in children, adolescents and adults. Pretoria; 2015. Available: http://www.health.gov.za/index.php/2014-03-17-09-09-38/policies-and-guidelines/category/230-2015p. Accessed 12 Aug 2015
102. Dryden-Peterson S, Lockman S, Zash R, Lei Q, Chen JY, Souda S, *et al.* Initial programmatic implementation of WHO Option B in Botswana associated with

increased projected MTCT. *Journal of Acquired Immune Deficiency Syndromes* 2015; **68**:245-249.
103. Kilewo C, Karlsson K, Ngarina M, Massawe A, Lyamuya E, Swai A, *et al.* Prevention of mother-to-child transmission of HIV-1 through breastfeeding by treating mothers with triple antiretroviral therapy in Dar es Salaam, Tanzania: the Mitra Plus study. *Journal of Acquired Immune Deficiency Syndromes* 2009; **52**:406-416.
104. Johnson LF. A model of paediatric HIV in South Africa. Cape Town: Centre for Infectious Disease Epidemiology and Research, University of Cape Town; 2010. Available: http://webdav.uct.ac.za/depts/epi/publications/documents/Paediatric_HIV_modelling5.pdf. Accessed 23 Feb 2011
105. Tenthani L, Haas AD, Tweya H, Jahn A, van Oosterhout JJ, Chimbwandira F, *et al.* Retention in care under universal antiretroviral therapy for HIV-infected pregnant and breastfeeding women ('Option B+') in Malawi. *AIDS* 2014; **28**:589-598.
106. Phillips T, Thebus E, Bekker LG, McIntyre J, Abrams EJ, Myer L. Disengagement of HIV-positive pregnant and postpartum women from antiretroviral therapy services: a cohort study. *Journal of the International AIDS Society* 2014; **17**:19242.
107. O'Donovan D, Ariyoshi K, Milligan P, Ota M, Yamuah L, Sarge-Njie R, *et al.* Maternal plasma viral RNA levels determine marked differences in mother-to-child transmission rates of HIV-1 and HIV-2 in The Gambia. *AIDS* 2000; **14**:441-448.
108. Coutsoudis A, Pillay K, Kuhn L, Spooner E, Tsai WY, Coovadia HM. Method of feeding and transmission of HIV-1 from mothers to children by 15 months of age: prospective cohort study from Durban, South Africa. *AIDS* 2001; **15**:379-387.
109. Goga AE, Van Wyk B, Doherty T, Colvin M, Jackson DJ, Chopra M. Operational effectiveness of guidelines on complete breast-feeding cessation to reduce mother-to-child transmission of HIV: results from a prospective observational cohort study at routine prevention of mother-to-child transmission sites, South Africa. *Journal of Acquired Immune Deficiency Syndromes* 2009; **50**:521-528.
110. Doherty T, Chopra M, Jackson D, Goga A, Colvin M, Persson LA. Effectiveness of the WHO/UNICEF guidelines on infant feeding for HIV-positive women: results from a prospective cohort study in South Africa. *AIDS* 2007; **21**:1791-1797.
111. National Breastfeeding Consultative Group. The Tshwane declaration of support for breastfeeding in South Africa. *South African Journal of Clinical Nutrition* 2011; **24**:214.
112. Goga AE, Jackson DJ, Singh M, Lombard C. Early (4-8 weeks postpartum) population-level effectiveness of WHO PMTCT option A, South Africa, 2012-2013. South African Medical Research Council and National Department of Health of South Africa; 2015.
113. Ashengo TA, Hatzold K, Mahler H, Rock A, Kanagat N, Magalona S, *et al.* Voluntary medical male circumcision (VMMC) in Tanzania and Zimbabwe: service delivery intensity and modality and their influence on the age of clients. *PLoS One* 2014; **9**:e83642.
114. Thirumurthy H, Masters SH, Rao S, Bronson MA, Lanham M, Omanga E, *et al.* Effect of providing conditional economic compensation on uptake of voluntary medical male circumcision in Kenya: a randomized clinical trial. *Journal of the American Medical Association* 2014; **312**:703-711.
115. Weiss SM, Zulu R, Jones DL, Redding CA, Cook R, Chitalu N. The Spear and Shield intervention to increase the availability and acceptability of voluntary medical male

circumcision in Zambia: a cluster randomised controlled trial *Lancet HIV* 2015; **2**:e181-e189.
116. Maughan-Brown B, Venkataramani AS, Nattrass N, Seekings J, Whiteside AW. A cut above the rest: traditional male circumcision and HIV risk among Xhosa men in Cape Town, South Africa. *Journal of Acquired Immune Deficiency Syndromes* 2011; **58**:499-505.
117. Mark D, Middelkoop K, Black S, Roux S, Fleurs L, Wood R, *et al.* Low acceptability of medical male circumcision as an HIV/AIDS prevention intervention within a South African community that practises traditional circumcision. *South African Medical Journal* 2012; **102**:571-573.
118. Liu A, Cohen S, Follansbee S, Cohan D, Weber S, Sachdev D, *et al.* Early experiences implementing pre-exposure prophylaxis (PrEP) for HIV prevention in San Francisco. *PLoS Medicine* 2014; **11**:e1001613.
119. Mugwanya KK, Donnell D, Celum C, Thomas KK, Ndase P, Mugo N, *et al.* Sexual behaviour of heterosexual men and women receiving antiretroviral pre-exposure prophylaxis for HIV prevention: a longitudinal analysis. *Lancet Infectious Diseases* 2013; **13**:1021-1028.
120. Grant RM, Anderson PL, McMahan V, Liu A, Amico KR, Mehrotra M, *et al.* Uptake of pre-exposure prophylaxis, sexual practices, and HIV incidence in men and transgender women who have sex with men: a cohort study. *Lancet Infectious Diseases* 2014; **14**:820-829.
121. Bekker LG, Johnson L, Cowan F, Overs C, Besada D, Hillier S, *et al.* Combination HIV prevention for female sex workers: what is the evidence? *Lancet* 2015; **385**:72-87.
122. Baeten JM, Donnell D, Ndase P, Mugo NR, Campbell JD, Wangisi J, *et al.* Antiretroviral prophylaxis for HIV prevention in heterosexual men and women. *New England Journal of Medicine* 2012; **367**:399-410.
123. Thigpen MC, Kebaabetswe PM, Paxton LA, Smith DK, Rose CE, Segolodi TM, *et al.* Antiretroviral preexposure prophylaxis for heterosexual HIV transmission in Botswana. *New England Journal of Medicine* 2012; **367**:423-434.
124. Marrazzo JM, Ramjee G, Richardson BA, Gomez K, Mgodi N, Nair G, *et al.* Tenofovir-based preexposure prophylaxis for HIV infection among African women. *New England Journal of Medicine* 2015; **372**:509-518.
125. Van Damme L, Corneli A, Ahmed K, Agot K, Lombaard J, Kapiga S, *et al.* Preexposure prophylaxis for HIV infection among African women. *New England Journal of Medicine* 2012; **367**:411-422.
126. Abdool Karim Q, Abdool Karim SS, Frohlich JA, Grobler AC, Baxter C, Mansoor LE, *et al.* Effectiveness and safety of tenofovir gel, an antiretroviral microbicide, for the prevention of HIV infection in women. *Science* 2010; **329**:1168-1174.
127. van der Straten A, Montgomery ET, Cheng H, Wegner L, Masenga G, von Mollendorf C, *et al.* High acceptability of a vaginal ring intended as a microbicide delivery method for HIV prevention in African women. *AIDS and Behavior* 2012; **16**:1775-1786.
128. McCormack S, Dunn DT, Desai M, Dolling DI, Gafos M, Gilson R, *et al.* Pre-exposure prophylaxis to prevent the acquisition of HIV-1 infection (PROUD): effectiveness results from the pilot phase of a pragmatic open-label randomised trial. *Lancet* 2015; **[In press]**.
129. South African National AIDS Council. National Strategic Plan for HIV Prevention, Care and Treatment for Sex Workers. Pretoria; 2013. Available:

http://www.sanac.org.za/resources/cat_view/7-publications/9-reports. Accessed 16 Oct 2014
130. Eisingerich AB, Wheelock A, Gomez GB, Garnett GP, Dybul MR, Piot PK. Attitudes and acceptance of oral and parenteral HIV preexposure prophylaxis among potential user groups: a multinational study. *PLoS One* 2012; **7**:e28238.
131. Mutua G, Sanders E, Mugo P, Anzala O, Haberer JE, Bangsberg D, *et al.* Safety and adherence to intermittent pre-exposure prophylaxis (PrEP) for HIV-1 in African men who have sex with men and female sex workers. *PLoS One* 2012; **7**:e33103.
132. Johnson LF, Dorrington RE, Bradshaw D, Coetzee DJ. The role of sexually transmitted infections in the evolution of the South African HIV epidemic. *Tropical Medicine and International Health* 2012; **17**:161-168.
133. Suraratdecha C, Ainsworth M, Tangcharoensathien V, Whittington D. The private demand for an AIDS vaccine in Thailand. *Health Policy* 2005; **71**:271-287.
134. Iman RL, Conover WJ. A distribution-free approach to inducing rank correlation among input variables. *Communications in Statistics. Simulation and Computation* 1982; **11**:311-334.
